# SLGCA: spatial cross-level graph contrastive autoencoder for multislice spatial domain identification and microenvironment exploration

**DOI:** 10.1093/bib/bbaf574

**Published:** 2025-11-03

**Authors:** Xin Lu, Murong Zhou, Guohua Wang, Qiaoming Liu, Yuming Zhao

**Affiliations:** College of Computer and Control Engineering, Northeast Forestry University, Harbin 150040, China; College of Life Science, Northeast Forestry University, Harbin 150040, China; College of Computer and Control Engineering, Northeast Forestry University, Harbin 150040, China; Department of Computer Science and Technology, Faculty of Computing, Harbin Institute of Technology, Harbin 150001, China; College of Artificial Intelligence, Henan University, Zhengzhou 450000, China; College of Computer and Control Engineering, Northeast Forestry University, Harbin 150040, China

**Keywords:** spatial transcriptomics, graph contrastive learning, spatial domain identification, multislice spatial domain identification

## Abstract

The development of spatial transcriptomics (ST) technologies has enabled researchers to better understand cells’ spatial organization and functional heterogeneity within their native tissue context. Spatial domain identification plays a crucial role in ST data analysis. However, most existing spatial domain identification methods do not fully exploit spatial information, and often fail to adequately integrate both local and global features, resulting in suboptimal spatial domain identification. We propose SLGCA, a novel method based on cross-level graph contrastive learning to address these challenges. SLGCA adopts a dual-channel learning mechanism, combining local-level contrastive learning based on spatial neighborhood information and global information contrastive learning across views, thereby significantly enhancing the accuracy of spatial domain identification. SLGCA can integrate multiple tissue sections without needing pre-alignment or external tools, eliminating batch effects and accurately identifying spatial domains across multiple slices. Experimental results show that SLGCA significantly outperforms the benchmark methods in spatial domain identification accuracy on ST data generated by multiple techniques. Moreover, SLGCA enables accurate dissection of tumor heterogeneity in human breast cancer datasets and effectively uncovers the heterogeneous tumor microenvironment in liver cancer, revealing two distinct fibroblast subtypes.

## Introduction

The advent of spatial transcriptomics (ST) technologies has enabled researchers to perform cell sequencing while preserving the spatial location information of cells, thereby providing deeper insights into the spatial structure and function of cells within tissues [1]. Currently, a variety of ST sequencing technologies have been developed, which can be broadly classified into two categories: array-based methods (such as 10× Visium [2] and Stereo-seq [3]) and imaging-based methods (such as osmFISH [4] and STARmap [5]). The former enables whole-transcriptome gene coverage but has relatively low spatial resolution. In contrast, the latter focuses on detecting specific genes and can achieve spatial resolution at the single-cell level. ST analysis relies heavily on the accuracy of spatial domain identification. Currently, mainstream spatial domain identification methods can be divided into two categories: nonspatial clustering methods and spatial clustering methods. Nonspatial clustering methods, such as K-means [6], Louvain [7], and Mclust [8], cluster data based solely on gene expression, ignoring spatial location information. This inevitably leads to poor spatial continuity and clustering accuracy.

In comparison, spatial methods significantly improve clustering accuracy by integrating spatial location information. For example, the representative algorithm, BayesSpace [9], uses Bayesian theory to combine gene expression data with spatial location information for clustering. However, BayesSpace struggles with complex spatial environments, such as ambiguous boundaries, due to the difficulty of machine learning methods in capturing nonlinear relationships in biological data. With the development of deep learning technologies, researchers have begun to apply deep learning to ST. For example, stLearn leverages deep learning to extract morphological features from histology images and spatial data, and normalizes gene expression using morphological distances for clustering analysis [10]. In addition, with the rise of graph neural networks (GNNs), ST data analysis has entered a new era [11]. This new paradigm has inspired numerous GNN-based methods. For example, SpaGCN combines histological images, gene expression, and spatial data, using graph convolutional networks (GCNs) to generate low-dimensional embeddings and applies unsupervised iterative clustering [12]. STAGATE further improves the integration of gene expression and spatial information using a graph attention network, thereby achieving more accurate clustering [13]. SEDR combines GCNs with variational autoencoders to learn more robust intermediate embeddings, which help identify more complex spatial domain patterns [14]. DenoiseST employs a dual-channel joint learning strategy based on GCNs and incorporates a zero-inflated negative binomial distribution to reconstruct and model ST data, further improving clustering accuracy [15].

Moreover, with the rise of self-supervised learning, methods based on contrastive learning have also emerged, aiming to enhance clustering performance by strengthening the model’s embedding representation capabilities. GraphST improves the learning ability of latent representations by introducing self-supervised graph contrastive learning, integrating gene expression, and spatial location information [16]. stDCL combines spatial-aware contrastive learning and clustering-feature contrastive learning to extract more discriminative low-dimensional latent representations from ST data [17]. CCST employs unsupervised GCNs to learn cell embeddings from graphs derived from ST data [18], and stGCL further extends multimodal integration by leveraging a histology-based visual transformer to encode tissue morphology and combining it with gene expression through a multimodal graph attention autoencoder (GATE) under a contrastive learning paradigm [19]. STMIGCL enhances clustering accuracy by constructing multiview graph convolutional neural networks and implicitly building contrastive learning frameworks [20]. stGRL, in contrast, adopts a local–local contrastive learning strategy coupled with a zero-inflated negative binomial distribution model to reconstruct the gene expression matrix, thereby enhancing the precision of spatial domain identification [21].

Although these methods have demonstrated strong performance in spatial clustering, they often underutilize spatial information. Typically, spatial relationships are incorporated only during the initial graph construction phase and are not explicitly leveraged during model training. This limits the ability of these methods to capture spatial relationships fully. Furthermore, many rely on a single paradigm, such as local–local or local–global, which restricts their capacity to model spatial hierarchies comprehensively [11]. For example, the local–local paradigm focuses on small-scale relationships but may overlook broader spatial patterns critical for understanding tissue structure [21]. Conversely, the local–global paradigm incorporates global context but may fail to capture fine-grained cellular interactions and hierarchical relationships in the tumor microenvironment (TME).

In ST, multislice spatial analysis is also crucial, as it integrates information from different tissue sections, providing a comprehensive understanding of tissue structure and microenvironmental heterogeneity. Several methods have been developed for this purpose. GraphST, e.g. aligns spatial coordinates across slices in advance and integrates them using GCNs combined with self-supervised contrastive learning. STAGATE relies on pre-aligned spatial coordinates and mitigates batch effects across slices by leveraging the shared nearest-neighbor algorithm with a predefined radius, enabling effective integration and spatial domain identification. stGCL constructs individual graphs for each slice to capture local spatial features and enhances cross-slice integration by performing contrastive learning within and across slices. In contrast, SEDR bypasses prior spatial alignment altogether by directly building a global graph structure across slices, enabling multislice spatial domain identification without additional preprocessing. Some methods also leverage external batch correction tools like Harmony to achieve cross-slice integration [22]. However, most existing approaches still require prior spatial alignment and tend to focus on either local or global structural modeling, making it challenging to capture both simultaneously. This often results in limitations in robustness and generalization of their contrastive learning strategies.

To overcome these challenges, we propose SLGCA, a novel deep learning framework based on GCNs that integrates local-level contrastive learning and global-level contrastive learning for single-slice and multislice spatial domain identification, uncovering tumor heterogeneity, and exploring the TME. SLGCA effectively combines gene expression data and spatial location information to learn latent embeddings that capture spatial heterogeneity and gene regulatory features. Unlike existing methods, SLGCA not only leverages spatial positions during graph construction in the preprocessing stage but also incorporates spatial information throughout the training process. Specifically, spatially adjacent cells are treated as positive pairs, encouraging their embeddings to be similar, while distant cells are designated as negative pairs, driving their embeddings apart. This mechanism enforces spatial constraints on the model at every training step, ensuring that spatial context continuously shapes the learned representations. By dynamically and iteratively integrating spatial information, SLGCA better captures spatial heterogeneity and latent regulatory structures within tissues, rather than relying solely on a one-time graph construction. At the global level, SLGCA aligns key feature dimensions across different views and suppresses redundancy, improving the expressiveness and discriminative power of the embeddings for clustering. This local and global contrastive learning combination enables SLGCA to uncover fine-grained spatial interactions and hierarchical tissue structures while capturing broader spatial relationships. Importantly, SLGCA supports end-to-end multislice spatial domain identification without requiring manual alignment or external batch correction tools. Its cross-level embedding contrast mechanism allows for automatic data integration across slices, simplifying the workflow, enhancing robustness, and enabling precise integration of gene expression and spatial location data across tissue sections.

We evaluated SLGCA on 17 tissue sections spanning multiple sequencing platforms. The results showed that SLGCA consistently outperformed 10 state-of-the-art methods in spatial domain clustering. SLGCA also demonstrated superior performance in multislice spatial domain identification. In a breast cancer dataset, it more accurately captured tumor heterogeneity, providing deeper insights into ST and supporting the development of targeted therapies. In liver cancer data, SLGCA successfully identified two distinct fibroblast subtypes, revealing new aspects of the TME.

## Materials and methods

### The workflow of SLGCA

**Figure 1:**
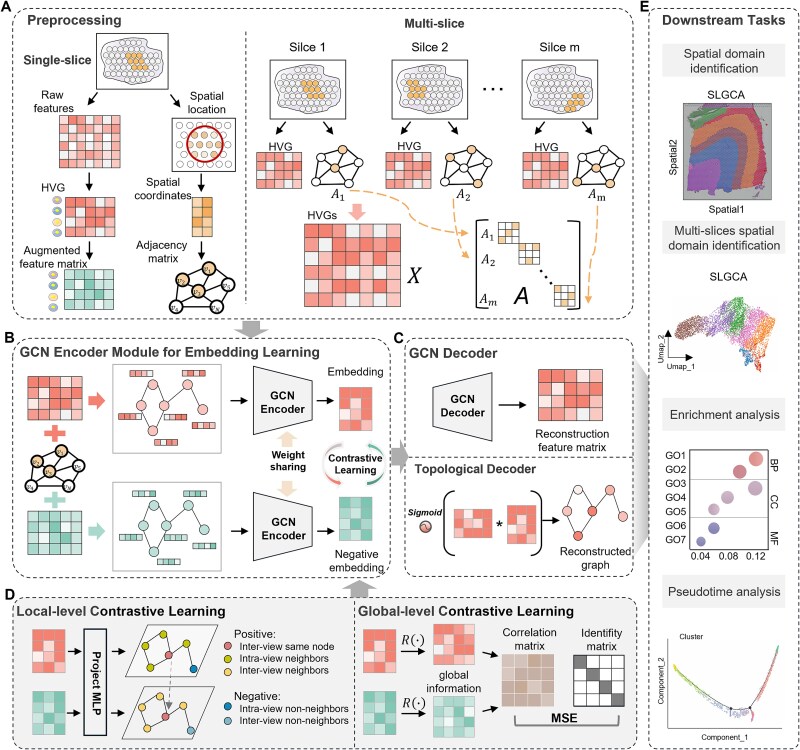
Overview of SLGCA. (A) The preprocessing stage consists of single and multislice procedures. In multislice preprocessing, the data augmentation strategy remains consistent with that used in the single slice setting. (B) SLGCA learns robust and discriminative intermediate embeddings by integrating a graph convolutional encoder with cross-level contrastive learning. (C) A GCN decoder and an inner product decoder are employed to reconstruct the gene expression and adjacency matrices, respectively. (D) Illustration of the local-level and global-level contrastive learning strategies: the former enhances local consistency and neighborhood discrimination, while the latter captures global semantics and suppresses redundant correlations. (E) SLGCA supports various downstream tasks, including single-slice and multislice spatial domain identification, enrichment analysis, and pseudotime analysis.

As shown in Fig. 1, the SLGCA framework takes the preprocessed gene expression matrix and spatial adjacency matrix as input. A contrastive view is generated through data augmentation of the original input, and both views are fed into a shared GCN encoder to obtain latent embeddings. SLGCA adopts a dual-channel cross-level contrastive learning strategy: local-level contrast captures relationships between nodes and their neighborhoods, while global-level contrast ensures structural consistency across the entire dataset. Following encoding, a symmetric decoder reconstructs the gene expression matrix, and an inner product decoder reconstructs the adjacency matrix. The model is trained using a combination of reconstruction and contrastive loss, ensuring effective integration of spatial and gene expression features. After training, the reconstructed gene expression matrix is reduced via PCA, and spatial domains are identified through clustering methods such as Mclust or Leiden.

### Datasets

The datasets used in this study are all publicly available. We provide the original data links in Supplementary File 1, Table S1, and upload them to the public database Zenodo (https://zenodo.org/records/15860656).

### Benchmarking methods

In this study, we systematically evaluated SLGCA against 10 state-of-the-art methods. These include: Mclust, which relies solely on gene expression; BayesSpace, which incorporates spatial information through machine learning; deep learning methods like stLearn and SpaGCN that integrate histological images, gene expression, and spatial data; GNN-based approaches such as STAGATE, SEDR, and DenoiseST; contrastive learning models like GraphST and stDCL that enhance feature representation via self-supervised learning; and STMIGCL, which uses a multiview neural network to model multimodal information. Some previously assessed methods were excluded from this comparison [23–25]. Detailed information on the benchmark experiments is provided in Supplementary File 2, Table S2.

### Data preprocessing

In this study, we denote the raw gene expression matrix from ST data as ${X}_{\mathrm{Raw}}\in{\mathbb{R}}^{N\times G}$, where $N$ represents the number of cells or spots, and $G$ denotes the number of genes. Due to the ST data’s high dimensionality and sparsity, directly using the full expression matrix as input may introduce substantial noise and incur significant memory overhead. To address this issue, we preprocess the raw expression matrix using the Scanpy toolkit to select highly variable genes (HVGs), resulting in a HVG expression matrix ${X}_{\mathrm{HVG}}\in{\mathbb{R}}^{N\times C}$, where $C$ is the number of HVGs. We then apply the logarithmic transformation and normalization on ${X}_{\mathrm{HVG}}$ to mitigate data skewness, following previous studies [16, 21, 26], and then standardize to ensure zero mean and unit variance. It is worth noting that we adopt a platform-specific gene selection strategy for different ST technologies: for high-throughput platforms such as 10× Genomics and Stereo-seq, we select the top 4000 most variable genes as input; whereas for technologies like osmFISH and STARmap, which detect a limited number of genes, we retain all available genes for analysis. This tailored gene selection strategy enhances the generalizability and adaptability of our method across diverse ST datasets. Ultimately, the input gene expression matrix used in the model is denoted as $X\in{\mathbb{R}}^{N\times M}$, where $N$ is the number of spots, and $M$ is the number of selected genes.

### Single-slice graph construction

For each tissue slice, we model it as an undirected neighborhood graph $G=\left(V,E\right)$, where $V=\left\{{v}_1,{v}_2,\cdots, {v}_N\right\}$, represents the set of $N$ spots, and $E\subseteq \left(V\times V\right)$ denotes the set of edges between these spots. The graph structure is represented by an adjacency matrix $A\in{\left\{0,1\right\}}^{N\times N}$, where ${A}_{ij}=1$ if spots ${v}_i$ is considered a neighbor of the spot ${v}_j$, and ${A}_{ij}=0$ otherwise. To accurately identify neighboring nodes, we calculate the Euclidean distances between spots and apply the K-nearest neighbors (KNN) algorithm to select the $K$ closest neighbors for each spot.

### Multislice graph construction

Each slice is processed independently in the initial steps when multiple tissue slices are integrated. We begin by applying a logarithmic transformation and normalization to each slice’s raw gene expression matrix, followed by selecting the top 5000 HVGs. We then extract the intersection of HVGs across all slices to obtain a unified set of shared HVGs, denoted as having $F$ genes. These shared HVGs are retained in each slice for the subsequent data integration step.

Assuming that there are *m* tissue slices, each containing ${N}_1,{N}_2,\cdots, {N}_m$​ spots, respectively, the total number of spots is $T=\sum_{i=1}^m{N}_i$. For each slice, we construct a corresponding adjacency matrix ${A}_1,{A}_2,\cdots, {A}_m$, using the same method described previously (e.g. KNN based on spatial coordinates).

To integrate the gene expression data, we concatenate the expression matrices of all slices along the row axis, forming a unified gene expression matrix denoted $X\in{\mathbb{R}}^{T\times F}$, where $T$ is the total number of spots, and $F$is the number of shared HVGs.

Next, we construct a block-diagonal adjacency matrix $\mathbb{A}$ by placing the adjacency matrices ${A}_1,{A}_2,\cdots, {A}_m$ along the diagonal, as shown in Equation (1):


(1) \begin{equation*} \mathbb{A}=\left[\begin{array}{ccc}{A}_1& \cdots & 0\\{}\vdots & \ddots & \vdots \\{}0& \cdots & {A}_m\end{array}\right]\in{R}^{T\times T} \end{equation*}

This block-diagonal matrix $\mathbb{A}$ preserves the local spatial relationships within each slice while ensuring no artificial connections are introduced between different slices.

In addition, during this process, we assign a slice identifier to each spot ${v}_i$, indicating which tissue slice it belongs to. This metadata can be used in downstream analysis for batch effect correction, domain adaptation, or visualization.

### Graph convolutional autoencoder

To effectively capture and integrate gene expression profiles and spatial information, we employ a graph convolutional autoencoder to learn latent representations from ST data. Specifically, the encoder and decoder are implemented using single-layer GCNs, with the normalized gene expression matrix $X$ and the spatial adjacency matrix $A$ as inputs. Encoder maps the input features into a latent representation $Z$, and decoder reconstructs the gene expression matrix $H$ from the latent representation $Z$. This process is defined as:


(2) \begin{equation*} Z=\sigma \left(\overset{\sim }{A}X{W}_e+{B}_e\right) \end{equation*}

(3) \begin{equation*} H=\sigma \left(\overset{\sim }{A}Z{W}_d+{B}_d\right) \end{equation*}

To enhance the stability of the graph convolution, we apply normalization to the adjacency matrix as shown in Equation (4):


(4) \begin{equation*} \overset{\sim }{A}={D}^{-\frac{1}{2}}\hat{A}{D}^{-\frac{1}{2}} \end{equation*}

where $\hat{A}=A+I$ is the adjacency matrix with added self-loops; $D={\sum}_j{A}_{ij}$ is the degree matrix; ${W}_e$ and ${W}_d$ are the trainable weight matrices in the encoder and decoder, respectively; ${B}_e$ and ${B}_d$ are the corresponding bias vectors for the encoder and decoder; and $\sigma \left(\cdot \right)$ denotes a nonlinear activation function (such as RELU).

To reconstruct the adjacency matrix $A$, we design an inner product decoder defined as:


(5) \begin{equation*} \hat{A}=\mathrm{sigmoid}\left({Z}^TZ\right) \end{equation*}

where $\mathrm{sigmoid}\left(\mathrm{x}\right)=1/\left(1+{\mathrm{e}}^{-x}\right)$ is a nonlinear activation function used to constrain the output within the range (0,1).

To simultaneously capture gene expression information and intercell relationships, we optimize the model by jointly minimizing the reconstruction errors of the gene expression matrix and the adjacency matrix. The total reconstruction loss is defined as:


(6) \begin{equation*} {l}_{\mathrm{rec}}=\frac{1}{N}\sum_{i=1}^N\left({\alpha}_1{\left\Vert{H}_i-{X}_i\right\Vert}^2+{\alpha}_2{\left\Vert{\hat{A}}_i-{A}_i\right\Vert}^2\right) \end{equation*}

where $N$ denotes the number of spots, $i$ denotes the $i$th spot, and ${\alpha}_1$ and ${\alpha}_2$ are weighting coefficients. According to experimental results, the model achieves optimal performance when ${\alpha}_1=1.0$ and ${\alpha}_2=0.5$.

### Local-level contrastive learning with spatial neighborhood information

Inspired by previous studies on contrastive learning [27], this study proposes a contrastive learning framework that emphasizes learning local information. The framework is designed to encourage the model to focus on nodes with similar contexts in the embedding space, enhancing the quality of the learned representations.

First, a corrupted graph $\overset{\sim }{G}$ is generated from the original graph $G$. Specifically, the rows of the original feature matrix $X$ (corresponding to the feature vectors of the nodes) are randomly shuffled while keeping the topological structure unchanged. This process randomly reassigns node feature vectors to different nodes, effectively redistributing them across the graph. Such a shuffling operation simulates potential noise or local feature perturbations that may occur in ST data, providing the model with an alternative perspective to learn feature patterns. For clarity, the original graph is referred to as ${G}_1$, and the corrupted graph as ${G}_2$.

In contrastive learning, the objective is to maximize the similarity between positive and negative pairs while minimizing the similarity between positive and negative pairs. Traditional contrastive learning approaches typically consider a node ${u}_i$ and its corresponding node ${v}_i$ in another view as a positive pair. In contrast, all other nodes are treated as negative samples [28, 29]. Therefore, the contrastive loss function for ${u}_i$ is defined as follows:


(7) \begin{align*} & l\left({u}_i\right)\nonumber\\ & =-\log \frac{e^{\theta \left({u}_i,{v}_i\right)/\tau }}{\underset{\mathrm{the}\ \mathrm{positive}\ \mathrm{pair}\ }{\underset{\Downarrow }{e^{\theta \left({u}_i,{v}_i\right)/\tau }}}+\underset{\mathrm{inter}-\mathrm{view}\ \mathrm{negative}\ \mathrm{pair}\mathrm{s}\ }{\underset{\Downarrow }{\sum_{k=1}^N{1}_{\left[k\ne i\right]}{e}^{\theta \left({u}_i,{v}_k\right)/\tau }}}+\underset{\mathrm{intra}-\mathrm{view}\ \mathrm{negative}\ \mathrm{pair}\mathrm{s}\ }{\underset{\Downarrow }{\sum_{k=1}^N{1}_{\left[k\ne i\right]}{e}^{\theta \left({u}_i,{u}_k\right)/\tau }}}} \end{align*}

where $\theta \left(\cdot \right)$ denotes cosine similarity, $\tau$ is a temperature parameter, and ${1}_{\left[k\ne i\right]}$ is an indicator function that equals 1 if $\left[k\ne i\right]$, and 0 otherwise.

However, this traditional contrastive learning approach inevitably overlooks the local neighborhood information of nodes. In ST data, spatially adjacent cells often exhibit similar gene expression profiles. Based on this assumption, we propose a neighborhood-aware contrastive learning paradigm. Specifically, when selecting positive pairs, we treat ${u}_i$ in ${G}_1$ as the anchor. The sources of positive pairs include: (i) the corresponding node ${v}_i$ in ${G}_2$; (ii) the neighbors of ${u}_i$ in ${G}_1$, where ${u}_j\in{N}_i^1$, and ${N}_i^1$​ denotes the neighborhood of the node ${u}_i$ in ${G}_1$; (iii) the neighbors of ${v}_i$ in ${G}_2$, where ${v}_j\in{N}_i^2$, and ${N}_i^2$denotes the neighborhood of the node ${v}_i$ in ${G}_2$. Therefore, the number of positive pairs for the anchor node ${u}_i$ is ${\hat{N}}_i={N}_i^1+{N}_i^2+1$, and all remaining nodes are treated as negative samples. The contrastive loss for the anchor ${u}_i$ is then defined as:


(8) \begin{equation*} l\left({u}_i\right)=-\log \frac{\left({f}^{1,2}\left(i,i\right)+\sum_{u_j\in{N}_i^1}{f}^{1,1}\left(i,j\right)+\sum_{v_j\in{N}_i^2}{f}^{1,2}\left(i,j\right)\right)/{\hat{N}}_i}{f^{1,2}\left(i,i\right)+{\sum}_{\left(j\ne i\right)}\left({f}^{1,1}\left(i,j\right)+{f}^{1,2}\left(i,j\right)\right)} \end{equation*}

where ${f}^{1,1}\left(i,j\right)={e}^{\theta \left({u}_i,{u}_j\right)/\tau }$，${f}^{1,2}\left(i,j\right)={e}^{\theta \left({u}_i,{v}_j\right)/\tau }$， and ${f}^{1,2}\left(i,i\right)={e}^{\theta \left({u}_i,{v}_i\right)/\tau }$. In this notation, “1,1” refers to intra-view (within the same graph), and “1,2” refers to inter-view (between graphs). The first term in the denominator corresponds to the positive pair of the same node across views, and the remaining terms can be further decomposed as:


(9) \begin{equation*} {\sum}_{\left(j\ne i\right)}{f}^{1,1}\left(i,j\right)=\underset{\mathrm{intra}-\mathrm{view}\ \mathrm{neighbor}\ \mathrm{pos}\ }{\underset{\Downarrow }{\sum_{u_j\in{N}_i^1}{f}^{1,1}\left(i,j\right)}}+\underset{\mathrm{intra}-\mathrm{view}\ \mathrm{neg}\ }{\underset{\Downarrow }{\sum_{u_j\notin{N}_i^1}{f}^{1,1}\left(i,j\right)}} \end{equation*}

(10) \begin{equation*} {\sum}_{\left(j\ne i\right)}{f}^{1,2}\left(i,j\right)=\underset{\mathrm{inter}-\mathrm{view}\ \mathrm{neighbor}\ \mathrm{pos}\ }{\underset{\Downarrow }{\sum_{v_j\in{N}_i^2}{f}^{1,2}\left(i,j\right)}}+\underset{\mathrm{inter}-\mathrm{view}\ \mathrm{neg}\ }{\underset{\Downarrow }{\sum_{v_j\notin{N}_i^2}{f}^{1,2}\left(i,j\right)}} \end{equation*}

Since the views are symmetric, the loss function remains unchanged when the anchor is set to ${v}_i$. The final loss function is defined as:


(11) \begin{equation*} {l}_{\mathrm{loc}-\cos }=\frac{1}{2N}\sum_{i=1}^N\left[l\left({u}_i\right)+l\left({v}_i\right)\right] \end{equation*}

### Global-level contrastive learning across views

Based on learning local features through neighborhood information, we propose a contrastive learning method for learning global features by aggregating local information into the global embedding. Specifically, after obtaining the intermediate embeddings $Z$, we introduce a readout function $R\left(\cdot \right)$, which aggregates the node embeddings into a global embedding via a weighted average. Similarly, the embedding $\hat{Z}$ from the other view generates the corresponding global embedding. The read function is defined as follows:


(12) \begin{equation*} R(Z)=\mathrm{\sigma} \left(\mathrm{normalize}\left(\frac{A\cdot Z}{\sum \left(A,\mathrm{axis}=1\right)}\right)\right) \end{equation*}

where $\mathrm{\sigma} \left(\cdot \right)$ represents a nonlinear sigmoid activation; $A\in{\mathbb{R}}^{N\times N}$ is the adjacency matrix, $Z\in{\mathbb{R}}^{N\times d}$ is the node embedding matrix, and $d$denotes the embedding dimension. Here, $\sum \left(A,\mathrm{axis}=1\right)$ represents the row-wise summation of *A*, i.e. the degree of each node, which ensures that the aggregation is normalized by the node degree.

Next, we compute the cosine similarity matrix $S\in{\mathbb{R}}^{d\times d}$ between the global embeddings of the two views, defined as:


(13) \begin{equation*} S=\cos \left(R(Z),R\left(\hat{Z}\right)\right) \end{equation*}

(14) \begin{equation*} \cos \left(X,Y\right)=\frac{X^T\cdot \mathrm{Y}}{{\left\Vert X\right\Vert}_2\cdot{\left\Vert Y\right\Vert}_2} \end{equation*}

We then use the mean squared error between $S$ and the identity matrix $I\in{\mathbb{R}}^{d\times d}$ as the contrastive loss function, defined as:


(15) \begin{equation*} {l}_{\mathrm{glo}-\cos }=\frac{1}{d^2}\sum_{i=1}^d\sum_{j=1}^d{\left({S}_{ij}-{I}_{ij}\right)}^2 \end{equation*}

here, ${S}_{ij}$ denotes the cosine similarity between the $i$th feature dimension in the first view and the $j$th feature dimension in the second view.

Global-to-global contrastive learning enhances the correlation between corresponding feature dimensions across views while suppressing redundant coupling between different feature dimensions. This effectively improves the expressiveness and discriminability of the latent representations, thereby enhancing clustering performance.

### Overall loss function

To enhance the model’s generalization capability, we adopt a joint optimization strategy throughout the training process, integrating the reconstruction loss, the local contrastive loss based on neighborhood information, and the global contrastive loss based on aggregated embeddings. The total objective loss function is defined as:


(16) \begin{equation*} \mathrm{Loss}=\alpha \ast{l}_{\mathrm{rec}}+\beta \ast{l}_{\mathrm{loc}-\cos }+\gamma \ast{l}_{\mathrm{glo}-\cos } \end{equation*}

where $\alpha$, $\beta,$ and $\gamma$ are weighting coefficients used to balance the contributions of each loss component. These are set to [10, 0.5, 0.5] by default.

### Clustering and refinement

After model training, we perform clustering analysis on the reconstructed representation matrix $H$ produced by the model. The steps are as follows: First, principal component analysis (PCA) is applied to reduce the dimensionality of $H$ to 20. If ground-truth labels are available, clustering is performed using the Gaussian Mixture Model-based mclust algorithm. If no labels are available, the Leiden clustering algorithm is used, with the resolution parameter set to 0.5.

To further refine the clustering results, we have adopted a postprocessing strategy consistent with baseline methods. Specifically, each spot’s neighborhood is defined as a circular region centered on the spot with a radius $r$ (default: 50). Each spot is then reassigned to the most frequent cluster label within its neighborhood.

Moreover, since the data have been merged into a single matrix during the preprocessing stage in multislice integration tasks, the cross-slice embeddings generated by the GNN can effectively eliminate batch effects. As a result, the spatial domain identification procedure is the same as that used for single-slice data.

### The overall architecture of SLGCA

In this study, we designed the encoder in the SLGCA architecture as a single-layer GCN with an embedding dimension of 64. The feature decoder mirrors the structure of the encoder, while the topology decoder is implemented as an inner product decoder. We employed a two-layer Multilayer Perceptron as the projection head to smooth the intermediate embeddings for the contrastive learning module. By default, the number of HVGs was set to 4000, the embedding dimension is set to 64, and the number of neighbors was set to 6. Model optimization was performed using the Adam optimizer with a learning rate 0.001 and no weight decay. The model was trained for 500 iterations. The default weights of the loss function, denoted as α, β, and γ, were set to 10, 0.5, and 0.5, respectively.

All experiments, including SLGCA and benchmark methods, were conducted on a server with an NVIDIA Tesla A100 GPU.

## Results

### SLGCA precisely identifies spatial domains in complex dorsolateral prefrontal cortex slices

To evaluate the ability of SLGCA to identify spatial domains in complex spatial tissue organization accurately, we applied it to a dataset of human dorsolateral prefrontal cortex (DLPFC) slices generated using the 10× Visium platform [2]. This dataset includes twelve annotated slices: eight slices labeled into seven regions (six cortical layers and one white matter region), and four slices labeled into five regions (four cortical layers and one WM region). SLGCA was compared against several baseline methods across all 12 slices.

**Figure 2:**
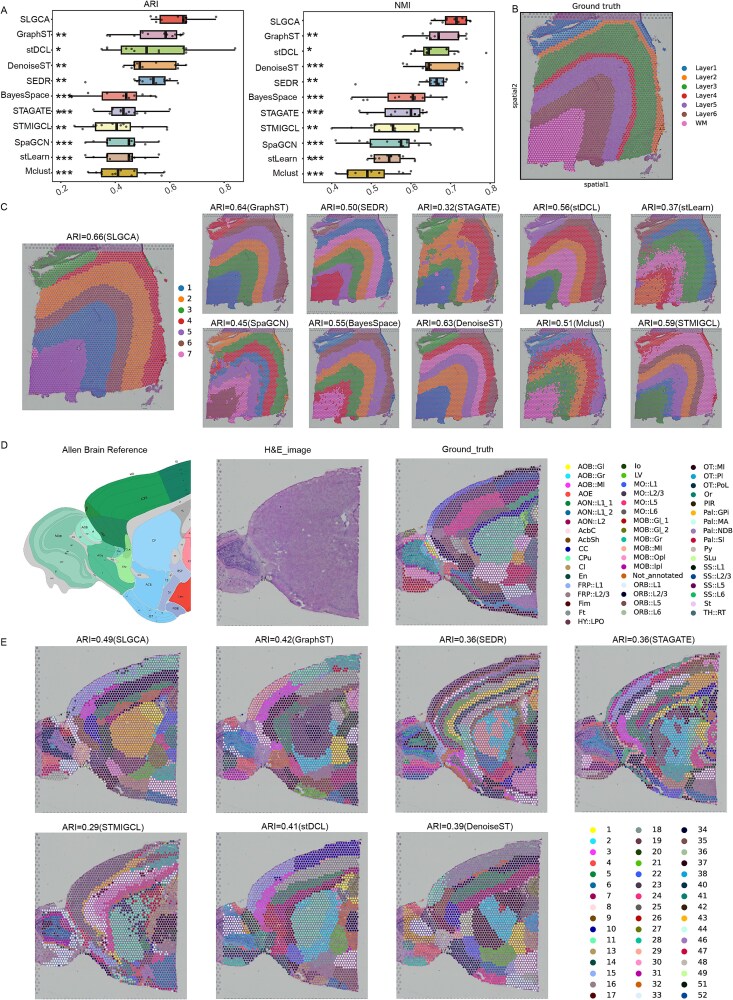
Experimental results of SLGCA on a complex brain tissue dataset. (A) Comparison of median ARI and NMI scores between SLGCA and baseline models on the DLPFC dataset. “^*^” indicates 0.01 ≤ *P* < .05; “^**^” indicates 0.001 ≤ *P* < .01; and “^***^” indicates *P* < .001. (B) Ground truth of slice no. 151673 in the DLPFC dataset. (C) Spatial domain identification results for SLGCA versus other baseline models on no. 151673. (D) The spatial domains of the mouse anterior brain dataset were manually annotated by the Allen mouse brain reference atlas. (E) Spatial clustering results of SLGCA and baseline methods on the mouse anterior brain dataset.

Figure 2A presents a performance comparison between SLGCA and baseline methods using ARI and NMI metrics. SLGCA consistently outperformed all other approaches, achieving the highest median ARI (0.65) and NMI (0.72), with mean scores of 0.63 and 0.71, respectively (Wilcoxon signed-rank test, FDR-adjusted $P<.05$ for all pairwise comparisons; see Supplementary File 3, Table S3). Compared to the second-best method, SLGCA improved ARI median and mean by 0.06 and 0.08, and NMI median and mean by 0.05 and 0.03, respectively. GraphST and stDCL also showed strong performance among the competitors, while SEDR delivered moderate but stable results. Mclust, lacking spatial information integration, performed the worst.

Next, we used slice no. 151673—widely adopted in previous studies [16, 23]—as a representative example to illustrate the performance of each method in spatial domain identification. Among all methods, SLGCA achieved the highest ARI score, followed by GraphST and DenoiseST. These three methods could clearly delineate seven regions that closely matched the ground truth annotations. In contrast, STAGATE and SpaGCN failed to distinguish all seven regions effectively, while stLearn, BayesSpace, and Mclust exhibited blurred boundaries between regions.

SLGCA accurately identified cortical layers within the DLPFC dataset and showed strong stability and robustness across multiple slices. Results for the remaining slices are provided in Supplementary File 4, Figs. S1–S3.

It is worth noting that in subsequent experiments, due to data format incompatibilities with some methods, we selected several top-performing methods—GraphST, SEDR, STAGATE, STMIGCL, stDCL, and DenoiseST—alongside SLGCA for further analysis.

### SLGCA accurately identifies spatial domains in a more complex mouse anterior brain dataset

To further validate the ability of SLGCA to identify spatial domains in complex tissue architectures, we applied it to the mouse anterior brain dataset, which features intricate spatial organization. This dataset was annotated concerning the Allen Brain Atlas [30], identifying 52 clusters (Fig. 2D).

As shown in Fig. 2E, SLGCA achieved the best performance again, with the highest ARI (0.49) and NMI (0.71), surpassing the second-best method by 0.07 and 0.02, respectively. GraphST and stDCL also performed well, with ARI scores of 0.42 and 0.41. In contrast, STMIGCL and STAGATE showed weaker performance, which was marked by fragmented spatial domains and indistinct boundaries. These results further support the effectiveness of SLGCA in resolving complex spatial structures. Notably, only SLGCA and stDCL successfully identified the “CPu” region, further highlighting their superior spatial resolution capabilities.

### SLGCA achieves accurate spatial domain identification across diverse spatial transcriptomics platforms

To validate the effectiveness of SLGCA in identifying spatial domains across datasets generated by different ST platforms, we evaluated SLGCA using three representative datasets derived from distinct sequencing technologies.

**Figure 3:**
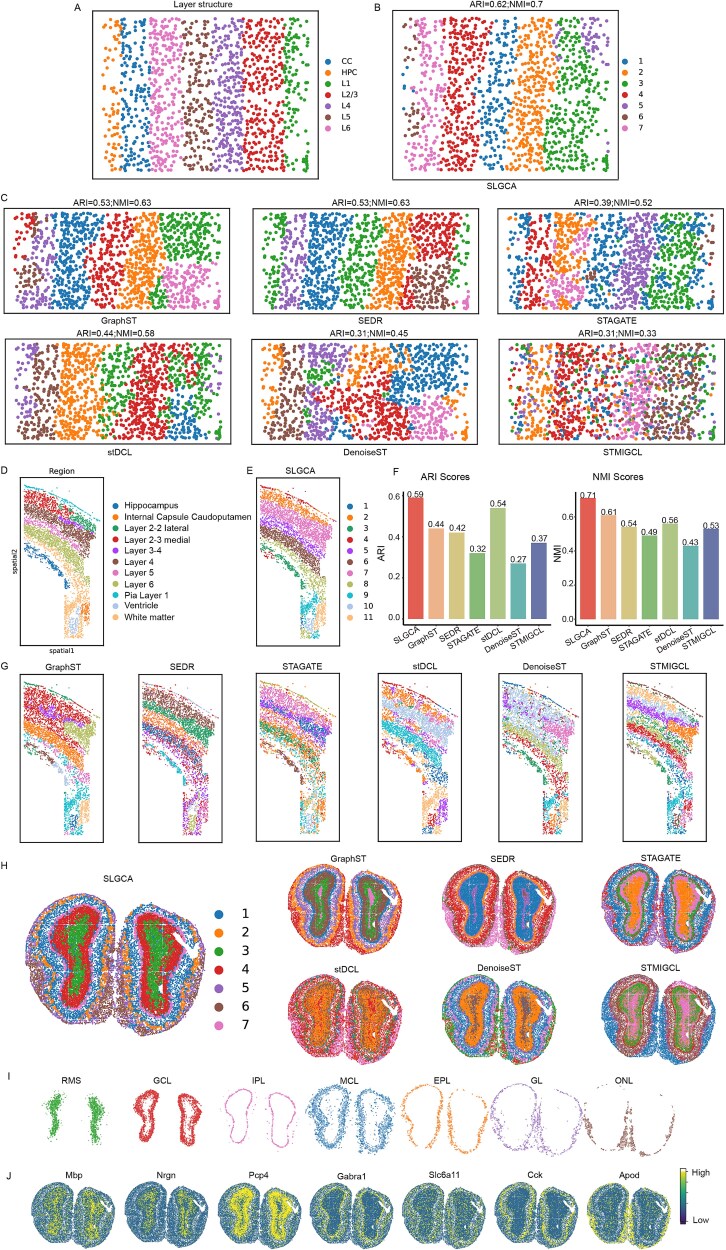
Experimental results of SLGCA on different platform datasets. (A) Ground truth labels for the mouse primary visual cortex (V1) dataset. (B) Clustering results of SLGCA for the mouse primary visual cortex (V1) dataset. (C) Clustering results of the benchmark experiment on the mouse primary visual cortex (V1) dataset. (D) Ground truth labels for the mouse somatosensory cortex dataset. (E) Cluster visualization of SLGCA on the mouse somatosensory cortex dataset. (F) Clustering results of SLGCA and benchmark methods on the mouse somatosensory cortex dataset. (G) Cluster visualization of benchmark experiments on the mouse somatosensory cortex dataset. (H) Clustering results of SLGCA and benchmark methods on the mouse factory bulb dataset. (I) Visualization results of SLGCA in the spatial domain. (J) Visualization results of marker genes on the mouse olfactory bulb dataset.

In the first experiment, we selected a mouse primary visual cortex (V1) dataset generated using the STARmap platform [5]. This dataset contains 1207 cells and 1020 genes, with ground truth labels shown in Fig. 3A. SLGCA was compared with several baseline methods on this dataset. As shown in Fig. 3B, SLGCA achieved the highest performance, with ARI and NMI scores of 0.62 and 0.70, respectively—improving over the second-best method by 0.09 (ARI) and 0.07 (NMI). GraphST and SEDR performed relatively well among the baselines but showed noticeable misclassifications. Other methods exhibited varying levels of domain mixing, with STMIGCL performing the worst, as its predicted domains poorly matched the ground truth (Fig. 3C). In the second experiment, we used a mouse somatosensory cortex dataset generated using the osmFISH platform [4]. This dataset comprises 4839 cells and 33 genes, with manual annotations in Fig. 3D. SLGCA again demonstrates excellent spatial domain recognition performance, with ARI and NMI scores of 0.59 and 0.71, respectively, which are 0.04 and 0.1 higher than the following best method (Figs. 3E and F). Only stDCL achieved an ARI above 0.5 among the baselines, while others exhibited evident domain mixing (Fig. 3G).

In the third experiment, we evaluated SLGCA on a widely used Stereo-seq-based mouse olfactory bulb dataset [3]. This dataset includes 19,109 cells and 14,376 genes. As shown in Fig. 3H, SLGCA effectively distinguished all seven cell types with well-defined region boundaries, although slight boundary ambiguity was observed in the MCL layer (Fig. 3I). GraphST achieved similar performance with even sharper boundaries, while all other methods failed to accurately separate the seven spatial domains (see Supplementary File 5, Fig. S4 for details). Furthermore, spatial staining based on known marker genes revealed that SLGCA’s clustering results were highly consistent with the spatial distributions of these markers (Fig. 3J). In addition to visual comparisons of spatial domains, we employed three unsupervised evaluation metrics—silhouette coefficient (SC), PAS, and CHAOS—to enhance the reliability of our assessment further. Among these metrics, higher SC values indicate better performance, while lower PAS and CHAOS values are preferable (see Supplementary File 5 for detailed results). Based on these results, although SLGCA did not achieve the highest score on every metric, it exhibited relatively balanced performance across all metrics. GraphST demonstrated the most consistent and robust performance among the baseline methods, which is also reflected in its spatial domain identification visualizations.

In summary, SLGCA performs excellently in the spatial domain identification across various datasets generated by different sequencing platforms. Whether applied to STARmap, osmFISH, or Stereo-seq—each varying in cell number, gene count, and spatial resolution—SLGCA consistently achieved accurate spatial segmentation and outperformed mainstream methods. Additionally, it showed strong stability and robustness, adapting effectively to diverse platforms and tissue types. These results highlight SLGCA’s broad applicability and superior capability in ST analysis.

### SLGCA enables accurate spatial domain identification across multislice spatial transcriptomics without prior alignment

SLGCA can perform spatial domain identification of multiple slices without prior spatial alignment. To comprehensively evaluate the effectiveness of slice integration, we introduced the Local Inverse Simpson’s Index (ILISI) [22], which reflects the degree of batch mixing, with higher values indicating better integration. In addition, since batch effect correction may lead to over-correction, we further employed ARI and NMI to evaluate clustering performance after batch removal, which also indirectly reflect the extent to which biological signals are preserved while eliminating batch variability. It is worth noting that stDCL, DenoiseST, and STMIGCL were excluded from this part of the evaluation, as slice integration was not addressed in their original publications.

**Figure 4:**
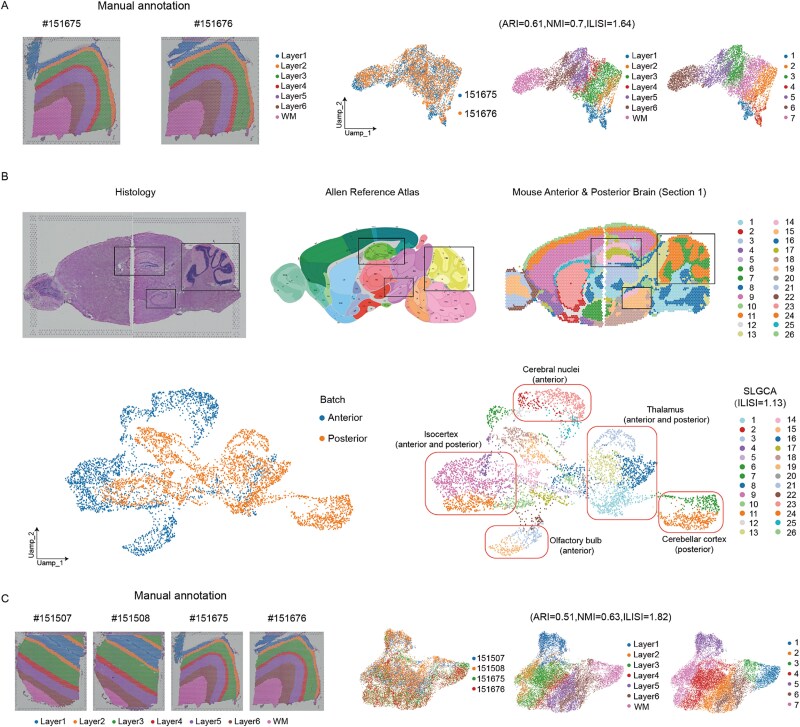
Multislice clustering performance of SLGCA. (A) Clustering performance of SLGCA on two vertical slices. (B) Clustering performance of SLGCA on two horizontal slices. (C) Clustering performance of SLGCA on two groups of slices from different sources.

We first evaluated SLGCA’s performance on two adjacent vertical slices (nos. 151675 and 151676) from the same sample in the DLPFC dataset. Their ground truth spatial domains are shown in Fig. 4A. After integration with SLGCA, clustering was performed similarly to the single-slice setting. UMAP visualization (Fig. 4A) demonstrates effective slice integration (ILISI = 1.64), with clustering results closely matching the ground truth (ARI = 0.61 and NMI = 0.7).

For multislice identification across horizontal slices, we tested anterior and posterior mouse brain regions. Unlike other methods requiring pre-alignment, SLGCA accurately recognized spatial tissue structures without spatial alignment (black box, Fig. 4B). Results aligned well with the Allen Brain Atlas and histology, showing strong integration (ILISI = 1.13), comparable to the best method. The integrated UMAP revealed the expected overlap patterns: some shared regions showed complete overlap (e.g. Isocortex and Thalamus), while region-specific domains remained distinctly separated (e.g. Olfactory bulb, Cerebellar cortex, and Cerebellar nuclei), indicating effective integration without over-mixing or over-separation (Fig. 4B, highlighted by red boxes). Other baseline methods, such as GraphST, SEDR, and STAGATE, also show similar UMAP distributions.

To evaluate the ability of SLGCA to integrate slices from the same region across different donors, we combined four slices from two DLPFC donors (nos. 151507, 151508, 151675, and 151676). Figure 4C shows their ground truth spatial domains and the UMAP embeddings after integration. SLGCA achieved effective integration (ILISI = 1.82), slightly lower than GraphST (ILISI = 1.96), while obtaining the highest clustering performance after integration (ARI = 0.51, NMI = 0.63). In addition, we also provide a comparison of UMAP visualization of the raw data without SLGCA processing and the UMAP visualization of the data after SLGCA processing (see Supplementary File 6, Fig. S5).

These results demonstrate that SLGCA can accurately identify spatial domains across multiple tissue sections, even without explicit spatial alignment. Notably, SLGCA consistently outperforms or matches state-of-the-art methods in both the quality of spatial domain identification and clustering accuracy (see Supplementary File 6, Figs. S6–S8). This highlights SLGCA’s robust ability to recognize coherent spatial patterns across vertically and horizontally arranged slices, underscoring its effectiveness in spatial domain detection in multislice ST data.

### SLGCA reveals distinct molecular features and spatial heterogeneity between ductal/lobular carcinoma *in situ* and invasive ductal carcinoma in breast cancer

**Figure 5:**
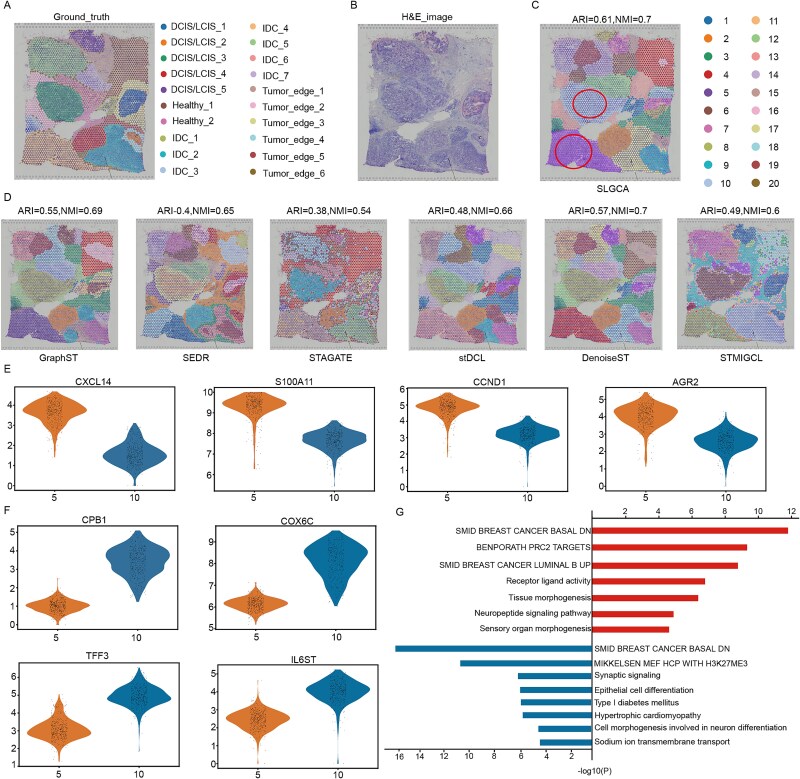
Experimental results of SLGCA on human breast cancer dataset. (A) True labels for the human breast cancer dataset. (B) H&E image of the human breast cancer dataset. (C) Clustering results of SLGCA. (D) Clustering results of benchmark methods. (E) Upregulated genes in spatial domain 5, shown as violin plots. (F) Upregulated genes in spatial domain 10, shown as violin plots. (G) the enrichment analysis results of spatial domains 5 (red) and 10 (blue).

This study analyzed a human breast cancer dataset annotated into 20 regions based on H&E staining and breast cancer-related marker genes [31]. These regions include invasive ductal carcinoma (IDC), ductal/lobular carcinoma *in situ* (DCIS/LCIS), healthy tissue, and tumor edge (Figs. 5A and B). Using SLGCA, we achieved the highest ARI (0.61) and NMI (0.7) scores among baseline methods, with clustering results shown in Fig. 5C. While DenoiseST and GraphST performed relatively well, STAGATE showed poorer results (Fig. 5D).

DCIS/LCIS, as early-stage noninvasive breast cancer, has significant potential to progress into IDC [32], making it critical to explore molecular differences. Focusing on Cluster 5 (IDC) and Cluster 10 (DCIS/LCIS), we identified 602 differentially expressed genes (*P*-value <0.05,$\left|\log\ \mathrm{fold}\ \mathrm{change}\right|$ > 2), revealing key molecular profiles.

In Cluster 5 (IDC), upregulated genes such as CXCL14, S100A11, CCND1, and AGR2 are linked to breast cancer progression, metastasis, and therapeutic resistance (Fig. 5E). For instance, CXCL14 may suppress tumor growth [33, 34], while S100A11 promotes cancer cell survival and migration [35, 36]. CCND1 drives oncogenic proliferation [37, 38], and AGR2 is a marker for metastatic potential [39, 40].

In Cluster 10 (DCIS/LCIS), upregulated genes such as CPB1, COX6C, TFF3, and IL6ST reflect early-stage tumor characteristics and immune regulation (Fig. 5F). CPB1 is associated with better survival outcomes and helps distinguish DCIS from IDC [41]. COX6C is linked to drug resistance [42], while TFF3 serves as a marker of differentiation and progression in breast cancer, particularly in DCIS, where it reflects a differentiated and secretory phenotype [43]. IL6ST (known as gp130) is involved in immune signaling and regulates local inflammation, cell survival, and differentiation within the TME [44, 45].

Further enrichment analysis of differentially expressed genes between Cluster 5 (IDC) and Cluster 10 (DCIS/LCIS) using Meatscape [46] revealed distinct biological processes and signaling pathways associated with each subtype (Fig. 5G). In Cluster 5 (IDC), pathways related to invasion and metastasis, such as M4960 (basal-like breast cancer genes) and M2410 (cancer metastasis), were significantly enriched, alongside processes like tissue morphogenesis and PRC2-mediated transcriptional repression. In contrast, Cluster 10 (DCIS/LCIS) is enriched in pathways like M2019 (H3K27ME3 epigenetic regulation), epithelial cell differentiation, and neurogenesis-related processes, consistent with a noninvasive phenotype. The complete enrichment analysis results are provided in the Supplementary File 7, Fig. S9.

In summary, SLGCA enables precise dissection of tumor heterogeneity, uncovering biologically meaningful spatial distinctions within breast cancer tissues. These findings deepen our understanding of tumor progression and inform the development of targeted therapies.

In addition, since the BRCA dataset has disputed annotations, we adopted the version labeled by Xu *et al.* [14]. Another version labeled by Long *et al.* [16] also exists, and we performed clustering analysis on this alternative annotation as well. The results were consistent with our original findings, as shown in Supplementary File 7, Fig. S10.

### SLGCA explores distinct fibroblast subtypes and functional diversity in the liver cancer tumor microenvironment

The heterogeneous TME is a vital aspect of tumor tissue. It comprises various nonmalignant cell types—such as fibroblasts, immune cells, and endothelial cells—that interact with malignant tumor cells, collectively influencing the hallmarks of cancer. TME is integral to tumor progression, therapeutic resistance, angiogenesis, and metastasis [47, 48].

**Figure 6:**
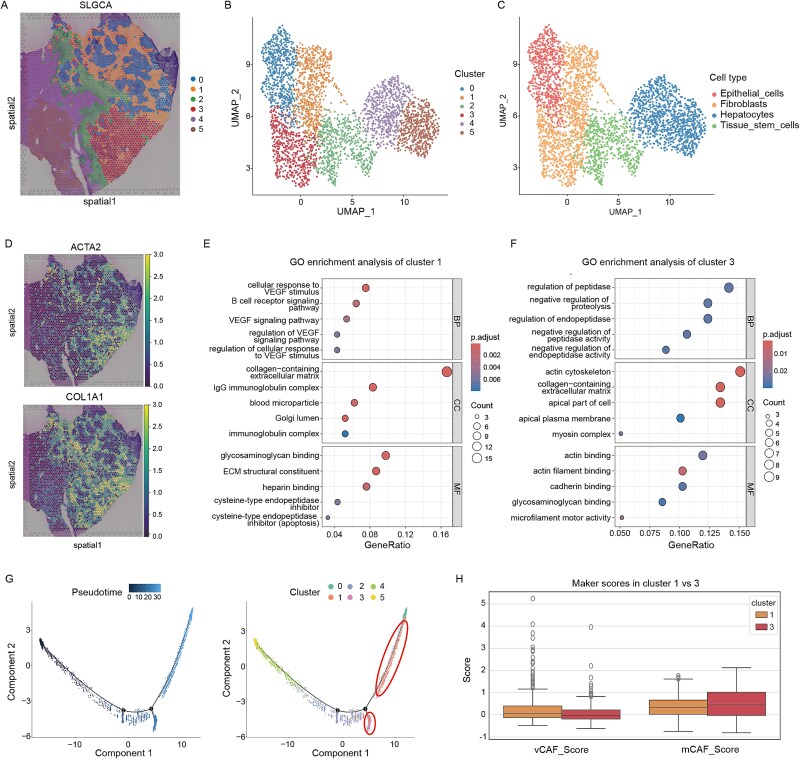
Experimental results of SLGCA on liver cancer dataset. (A) clustering results of SLGCA. (B) Display clustering results with UMAP. (C) Cell type annotation results using SingleR. (D) Fibroblast marker gene visualization. (E) GO enrichment analysis results of Cluster 1. (F) GO enrichment analysis results of Cluster 3. (G) Pseudo-timing analysis visualization. (H) The marker gene set scores of Clusters 1 and 3.

In this study, we analyzed ST data from a liver cancer patient using the 10× Visium platform [49]. We applied the Leiden algorithm without ground-truth cell labels for unsupervised clustering, identifying six groups (Fig. 6A and B). Using SingleR [50] and marker gene visualization, we annotated four cell types: epithelial cells, fibroblasts, hepatocytes, and tissue stem cells, with Clusters 1 and 3 identified as fibroblasts (Fig. 6C and D). Fibroblasts, particularly cancer-associated fibroblasts (CAFs), are key components of the TME, contributing to tumor progression through interactions with malignant cells, angiogenesis, immune cell recruitment, structural regulation, and migration facilitation [51]. Given their importance, we focused on Clusters 1 and 3 to investigate fibroblast subpopulation heterogeneity.

We first analyzed differential gene expression between the cells in Clusters 1 and 3, identifying 283 differentially expressed genes (*P*-value <.05，|log fold change| > 2). We further conducted Gene Ontology (GO) enrichment analysis [52] based on these genes to explore the functional differences between these two fibroblast subpopulations. Cluster 1 fibroblasts were enriched in biological processes such as “cellular response to vascular endothelial growth factor stimulus,” “B cell receptor signaling pathway,” and “vascular endothelial growth factor signaling pathway,” suggesting roles in angiogenesis and immune regulation. At the cellular component (CC) level, these cells were associated with “collagen-containing extracellular matrix,” “immunoglobulin complex,” and “blood microparticle,” highlighting their involvement in extracellular matrix stability and inflammatory responses. For molecular function (MF), they were enriched in “extracellular matrix structural constituent,” “glycosaminoglycan binding,” and “cysteine-type endopeptidase inhibitor activity,” indicating anti-fibrotic potential through matrix degradation suppression (Fig. 6E).

Cluster 3 fibroblasts were enriched in processes related to “regulation of peptidase activity,” “negative regulation of proteolysis,” and “cytoskeletal remodeling,” implying roles in matrix remodeling and anti-inflammatory functions. Cellular component enrichment included “actin cytoskeleton,” “collagen-containing extracellular matrix,” and “myosin complex,” reflecting dynamic cytoskeletal regulation and enhanced contractility. At the MF level, they were enriched in “actin filament binding,” “microfilament motor activity,” and “cadherin binding,” supporting functions in cell migration, adhesion, and tissue reconstruction (Fig. 6F).

Trajectory analysis revealed that Cluster 1 fibroblasts were located near terminally differentiated regions, enriched in pathways related to angiogenesis, immune regulation, and extracellular matrix remodeling, suggesting roles in tissue repair and vascular support (Fig. 6G). In contrast, Cluster 3 fibroblasts were distributed at branching points, enriched in cytoskeletal remodeling, protease regulation, and cell motility functions, indicating a more intermediate state geared toward tissue remodeling, early wound repair, and fibrotic processes.

Finally, we manually curated gene sets associated with different CAF subtypes and used them to score Clusters 1 and 3. The results revealed significant differences in vCAF and mCAF subtype scores between the two clusters: Cluster 1 showed higher scores for the vCAF gene set, while Cluster 3 had higher scores for the mCAF gene set (Fig. 6H). According to previous studies, the vCAF subtype is typically associated with angiogenesis, maintenance of vascular structure, and interactions with endothelial cells. In contrast, the mCAF subtype is primarily involved in the synthesis, remodeling, and degradation of the extracellular matrix, thus regulating the structural organization of the tumor and facilitating cell migration. These findings are highly consistent with our previous analyses [53].

In summary, Cluster 1 fibroblasts exhibit a vascular-associated phenotype, contributing to tumor angiogenesis and vascular support, whereas Cluster 3 fibroblasts display a matrix-associated phenotype, regulating extracellular matrix remodeling and tissue reconstruction. These findings further validate the effectiveness of SLGCA in dissecting cellular heterogeneity within the TME.

### Evaluation metrics for SLGCA

We used ARI and NMI to evaluate clustering performance, and ILISI to assess ST data integration quality in slice alignment experiments. For unlabeled datasets, we also introduce three unsupervised clustering metrics, SC, PAS, and CHOAS, to evaluate the clustering performance. See Supplementary File 8 for details.

### Robustness and efficiency analysis of SLGCA

We systematically evaluated the contributions of the local and global contrastive learning modules in SLGCA through ablation experiments. The results demonstrated that both modules significantly enhance model performance—removing either module led to a slight decline, while removing both resulted in a substantial drop. In addition, we conducted ablation experiments on the topological decoder, which also proved to play an essential role within the overall model architecture. We further performed hyperparameter sensitivity analyses, showing that the model achieves optimal performance when using 4000 HVGs, four neighbors, and loss function weights of $\alpha =10$ and $\beta =\gamma =0.5$. Moreover, we assessed SLGCA’s runtime and memory usage on two representative datasets, demonstrating its strong computational efficiency and scalability across different data scales and platforms. We also discussed the impact of graph construction strategies and downstream clustering algorithms. In addition, we evaluated the performance of using the learned intermediate embeddings as input for clustering. Detailed information is provided in Supplementary File 9.

### Detailed description of the downstream application design process

We have summarized the downstream applications of SLGCA and the functions and packages used in this study. Detailed information can be found in Supplementary File 10.

### Differences from other contrastive learning methods

To further highlight the innovations of SLGCA and distinguish it from other contrastive learning-based approaches, we compared two representative methods, GraphST and stGCL. We conducted a detailed analysis across three key aspects: model architecture, contrastive learning mechanism, and loss function design. This comparative study underscores the unique advantages and innovations of SLGCA. See the Supplementary File 11 for details.

## Discussion

Spatial domain identification is critical for analyzing ST data, as its accuracy directly impacts biological interpretations and downstream analyses. To address this challenge, we propose SLGCA, an innovative framework leveraging cross-level contrastive learning to improve the accuracy and generalizability of spatial domain identification. Systematic evaluations across 17 tissue slices from diverse sequencing platforms demonstrate that SLGCA consistently outperforms leading baseline methods, accurately identifying spatial domains characterized by coherent gene expression patterns.

In multislice analysis, SLGCA effectively integrates spatial data from different tissue sections, excelling at cross-slice spatial domain identification without requiring prior spatial alignment or external tools. Its innovative contrastive learning mechanism enables automatic recognition and alignment of biologically consistent spatial domains across vertical and horizontal slices, achieving efficient integration and high accuracy.

Applied to human breast cancer data, SLGCA revealed molecular and spatial heterogeneity between IDC and DCIS/LCIS, offering insights into tumor progression and targeted therapy development. Two fibroblast subtypes with distinct functions were identified in liver cancer, showcasing its strength in dissecting TME heterogeneity.

We also evaluated SLGCA’s robustness, parameter sensitivity, and computational efficiency, providing practical user guidance. However, incorporating spatial neighborhood information in local contrastive learning increases computational and memory costs, particularly for large-scale datasets, highlighting areas for future optimization.

With the advancement of spatial multi-omics technologies, integrating multiple omics layers within the same spatial context has become essential for understanding tissue microenvironments. Developing analytical frameworks capable of processing such data will be a key focus.

## Conclusion

SLGCA is an effective and dependable tool for spatial domain identification, yielding accurate results from data produced by various sequencing technologies and enabling the integration of multiple tissue slices. As ST continues to evolve and data volumes increase, SLGCA is well positioned to enhance our understanding of tissue microenvironments and to provide strong support for studies on disease mechanisms and precision medicine.

**Key Points**

- Proposed a cross-level contrastive learning framework combining local contrastive learning with spatial neighborhood information and global structural contrastive learning to enhance spatial domain identification.
- Achieves end-to-end multislice spatial domain identification, automatically aligning biologically consistent domains across tissue slices without prior alignment or external tools.
- Demonstrated strong performance in tumor microenvironment exploration, revealing molecular heterogeneity in breast and liver cancer datasets.

## Supplementary Material

Final-Supplementary_Files_bbaf574

## Data Availability

The datasets used in this study are all publicly available. We provide the original data links in Supplementary File 1, Table S1, and upload them to the public database Zenodo (https://zenodo.org/records/15860656). The source code for SLGCA can be downloaded from GitHub (https://github.com/luxin-heart/SLGCA).
