# Supplementary material for: SLGCA: spatial cross-level graph contrastive autoencoder for multislice spatial domain identification and microenvironment exploration
Source: Brief Bioinform. 2025 Nov 3;26(6):bbaf574. doi: 10.1093/bib/bbaf574 (PMC12581855; doi:10.1093/bib/bbaf574)
Supplement: Final-Supplementary_Files_bbaf574 [file final-supplementary_files_bbaf574.docx]

**Supplementary file for SLGCA: Spatial Cross-Level Graph Contrastive Autoencoder for multi-slice spatial domain Identification and microenvironment exploration**

Xin Lu^1†^, Murong Zhou^2†^, Guohua Wang^1,3^*, Qiaoming Liu^4^*, Yuming Zhao^1^*

**Supplementary File 1：Dataset**

Table S1. Description of all ST datasets used in this study

| **Platform** | **Tissue** | **Section** | **Spots** | **Genes** | **URLs** |
| --- | --- | --- | --- | --- | --- |
| 10 x Visium | DLPFC | 151507 | 4226 | 33538 | http://research.libd.org/spatialLIBD/ |
|  |  | 151508 | 4384 |  |  |
|  |  | 151509 | 4789 |  |  |
|  |  | 151510 | 4634 |  |  |
|  |  | 151669 | 3661 |  |  |
|  |  | 151670 | 3498 |  |  |
|  |  | 151671 | 4110 |  |  |
|  |  | 151672 | 4015 |  |  |
|  |  | 151673 | 3639 |  |  |
|  |  | 151674 | 3673 |  |  |
|  |  | 151675 | 3592 |  |  |
|  |  | 151676 | 3460 |  |  |
|  | Mouse anterior brain | None | 2695 | 32285 | https://doi.org/10.5281/zenodo.10968451. |
| STARmap | Mouse primary visual cortex | None | 1207 | 1020 | https://sysbio.gzzoc.com/Mouse-Brain-Aging/ |
| osmFISH | Mouse somatosensory cortex | None | 4,839 | 33 | https://github.com/JinmiaoChenLab/SEDR_analyses/tree/master/data |
| Stereo-seq | Mouse olfactory | None | 19109 | 14375 | https://github.com/JinmiaoChenLab/SEDR_analyses/tree/master/data |

Table S1. Description of all ST datasets used in this study(continued)

| **Platform** | **Tissue** | **Section** | **Spots** | **Genes** | **URLs** |
| --- | --- | --- | --- | --- | --- |
| 10 x Visium | Mouse Brain Section 1 (SagittalAnterior) | None | 2695 | 32285 | https://doi. org/10.5281/zenodo.10277127 |
| 10 x Visium | Mouse Brain Section 1 (SagittalPosterior) | None | 3355 | 32285 | https://doi.org/10.5281/zenodo.10968451. |
| 10 x Visium | Human Breast Cancer | None | 3798 | 36601 | https://support.10xgenomics.com/spatial-gene-expression/datasets |

**Supplementary File 2：Benchmarking methods**

Table S2. Description of all benchmarking methods

| **Method** | **Platform** | **Year** | **Github** |
| --- | --- | --- | --- |
| Mclust | R | 2002 | https://mclust-org.github.io/mclust/ |
| BayesSpace | R | 2021 | https://github.com/edward130603/BayesSpace |
| stLearn | Python | 2020 | https://github.com/BiomedicalMachineLearning/stLearn |
| SpaCGN | Python | 2021 | https://github.com/jianhuupenn/SpaGCN |
| STAGATE | Python | 2022 | https://github.com/zhanglabtools/STAGATE |
| SEDR | Python | 2024 | https://github.com/JinmiaoChenLab/SEDR |
| DenoiseST | Python | 2024 | https://github.com/cuiyaxuan/DenoiseST/tree/master |
| GraphST | Python | 2024 | https://github.com/JinmiaoChenLab/SEDR |
| stDCL | Python | 2025 | https://github.com/Philyzh8/stDCL |
| STMIGCL | Python | 2025 | https://github.com/YuBinLab-QUST/STMIGCL/ |

**Supplementary File 3：Statistical comparison of clustering performance between SLGCA and baseline methods**

We comprehensively evaluated SLGCA’s clustering performance against state-of-the-art benchmark methods on the **DLPFC dataset**, using Wilcoxon signed-rank tests to assess statistical significance. We further applied Benjamini–Hochberg correction for multiple testing to ensure rigor. Table S3 reports both raw and FDR-adjusted p-values comparing SLGCA with ten benchmark methods. The results show that SLGCA consistently outperforms all competing approaches in ARI and NMI metrics (FDR-adjusted p < 0.05). Notably, for the majority of methods (including STAGATE, stLearn, SpaGCN, BayesSpace, and Mclust), the adjusted p-values reached highly significant levels (FDR-adjusted p < 0.001), providing strong evidence for the superior performance of SLGCA in spatial domain identification. These findings validate the robustness of our approach and highlight SLGCA as a more precise computational tool for spatial transcriptomics analysis.

Table S3. Raw and FDR-adjusted Wilcoxon test p-values comparing SLGCA with benchmark methods on the DLPFC dataset

| **SLGCA vs.** | **ARI** | **ARI(adj.p)** | **NMI** | **NMI(adj.p)** |
| --- | --- | --- | --- | --- |
| GraphST | 2.9297e-03 | 3.2237e-03 | 9.7656e-04 | 1.4648e-03 |
| SEDR | 9.7656e-04 | 1.4648e-03 | 1.4648e-03 | 1.4648e-03 |
| STAGATE | 4.8828e-04 | 9.7656e-04 | 4.8828e-04 | 9.7656e-04 |
| stDCL | 2.2461e-02 | 2.4967e-02 | 1.5137e-02 | 1.6819e-02 |
| stLearn | 4.8828e-04 | 9.7656e-04 | 4.8828e-04 | 9.7656e-04 |
| SpaGCN | 4.8828e-04 | 9.7656e-04 | 4.8828e-04 | 9.7656e-04 |
| BayesSpace | 4.8828e-04 | 9.7656e-04 | 4.8828e-04 | 9.7656e-04 |
| DenoiseST | 9.7656e-04 | 1.4648e-03 | 4.8828e-04 | 9.7656e-04 |
| Mclust | 4.8828e-04 | 9.7656e-04 | 4.8828e-04 | 9.7656e-04 |
| STMIGCL | 9.7656e-04 | 1.4648e-03 | 1.4648e-03 | 1.4648e-03 |

**Supplementary File 4：Experimental results of SLGCA and baseline methods on DLPFC dataset**

**
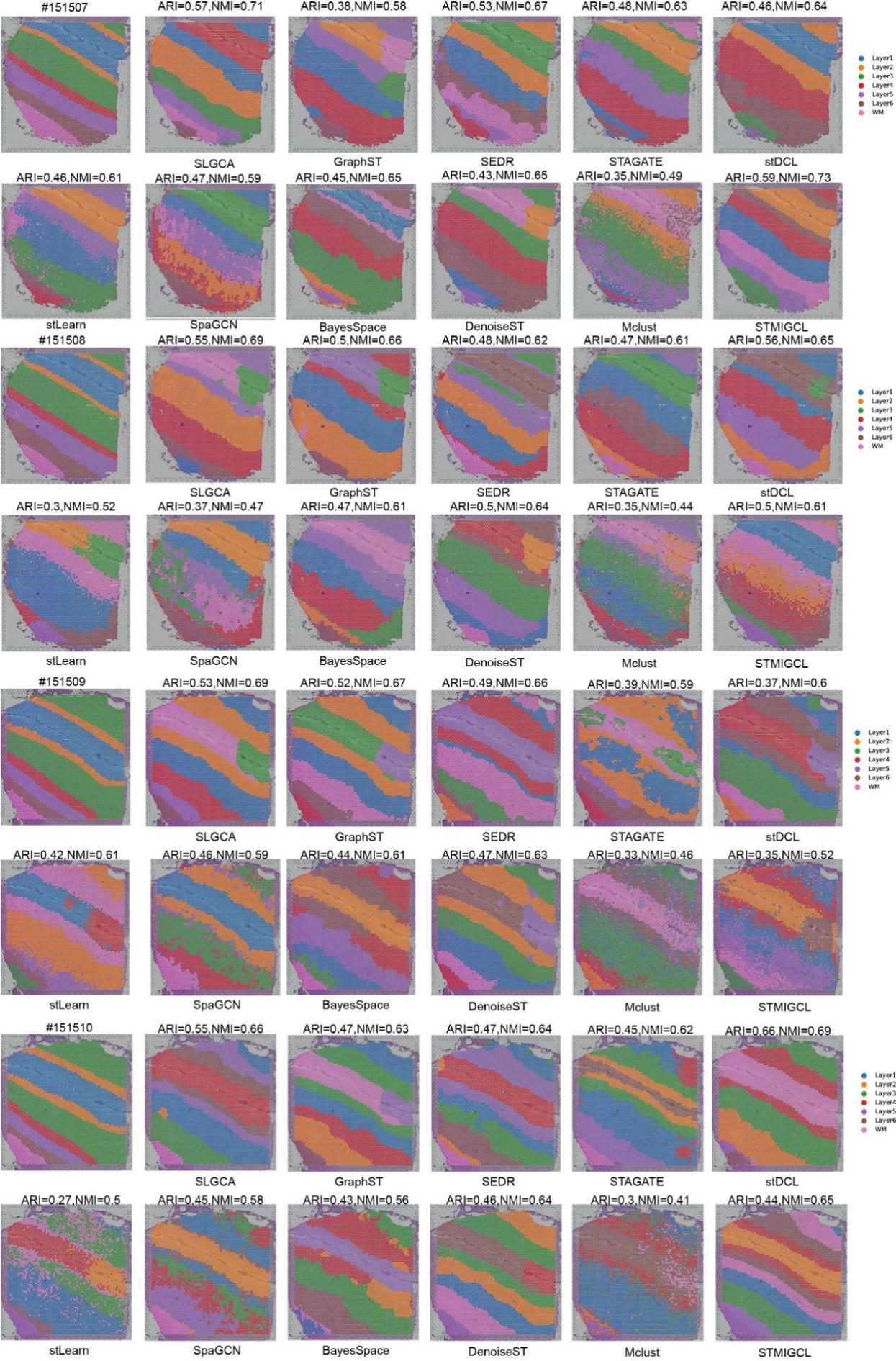
**

Figure S1. #151507~#151510

**
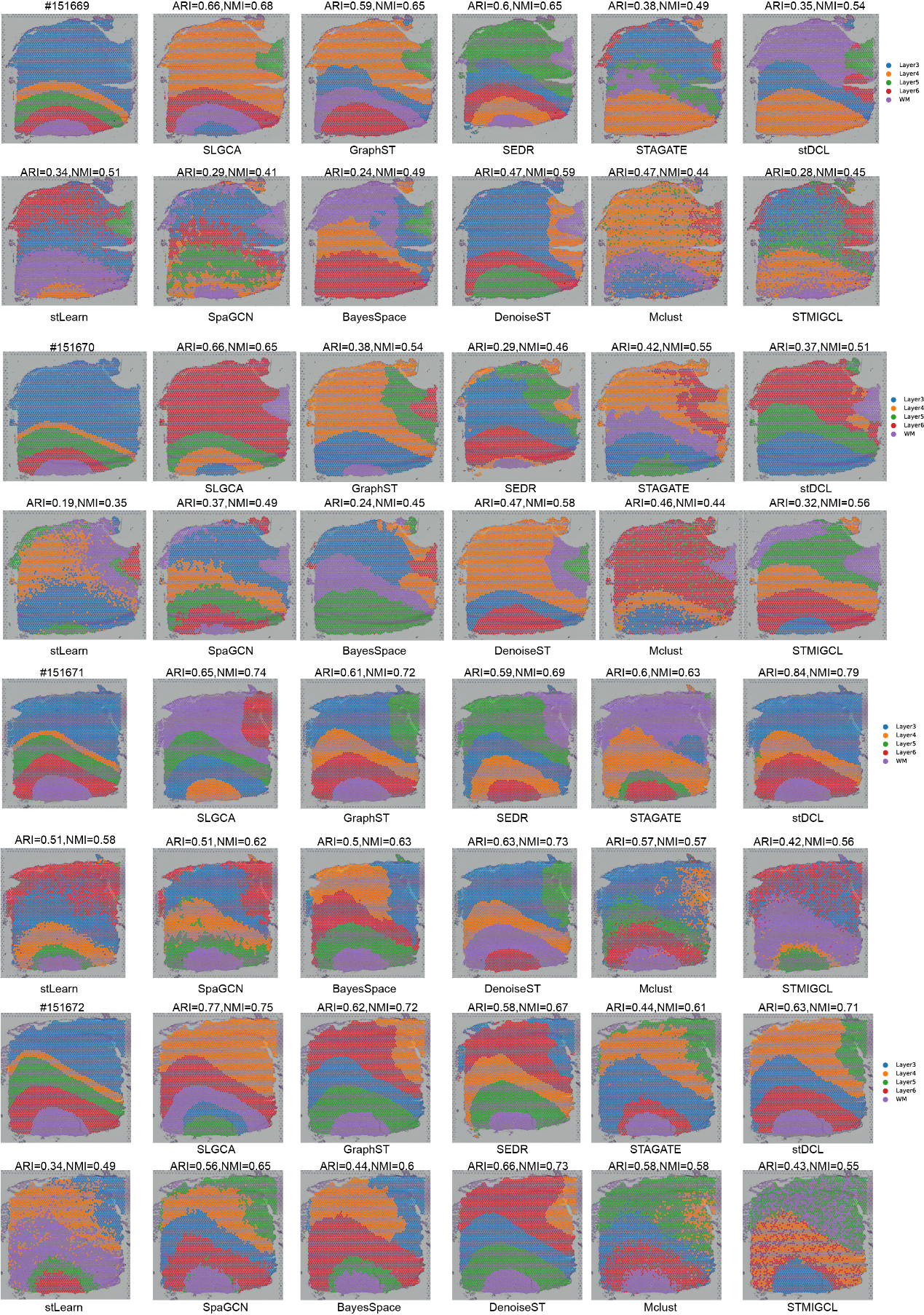
**

Figure S2. #151669~#151672

**
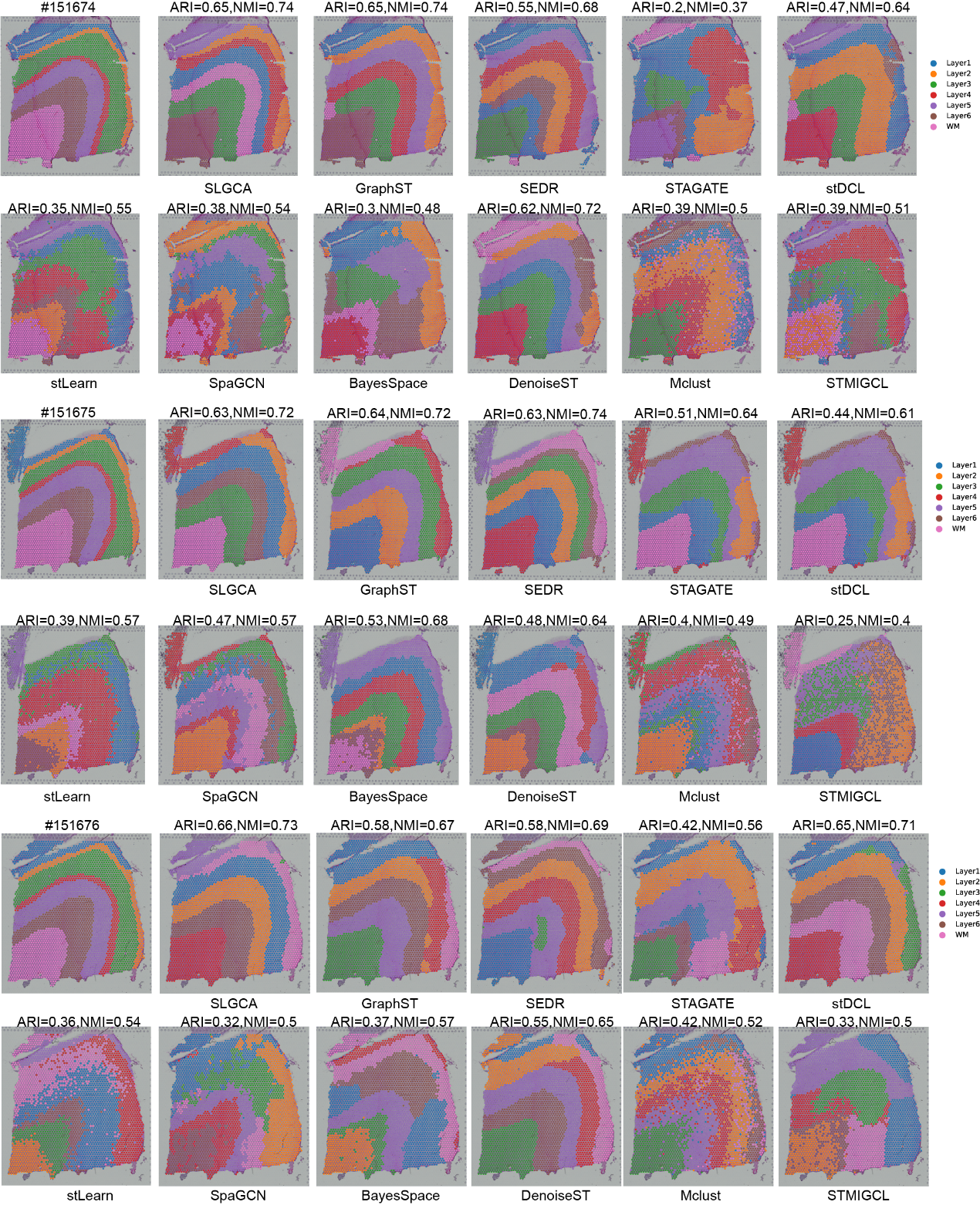
**

Figure S3. #151674~#151676

**Supplementary File 5：Clustering performance of benchmark experiments on mouse olfactory bulb dataset**

In addition to comparing the spatial domain visualizations, we further incorporated three unsupervised evaluation metrics to enhance the reliability of our comparisons. These metrics are the silhouette coefficient (SC), PAS, and CHAOS. Higher SC values indicate better performance, whereas lower PAS and CHAOS values are preferred. The specific metric scores of SLGCA and the benchmark methods are summarized in the table below:

Table S4. Unsupervised metrics of SLGCA and baseline methods on the mouse factory bulb dataset

| **Method** | **SC** | **PAS** | **CHAOS** |
| --- | --- | --- | --- |
| SLGCA | 0.07 | 0.11 | 0.04 |
| STAGATE | 0.16 | 0.1 | 0.04 |
| SEDR | -0.09 | 0.03 | 0.05 |
| GraphST | 0.08 | 0.07 | 0.03 |
| stDCL | 0.09 | 0.29 | 0.18 |
| DenoiseST | 0.07 | 0.17 | 0.07 |
| STMIGCL | -0.03 | 0.22 | 0.13 |

**
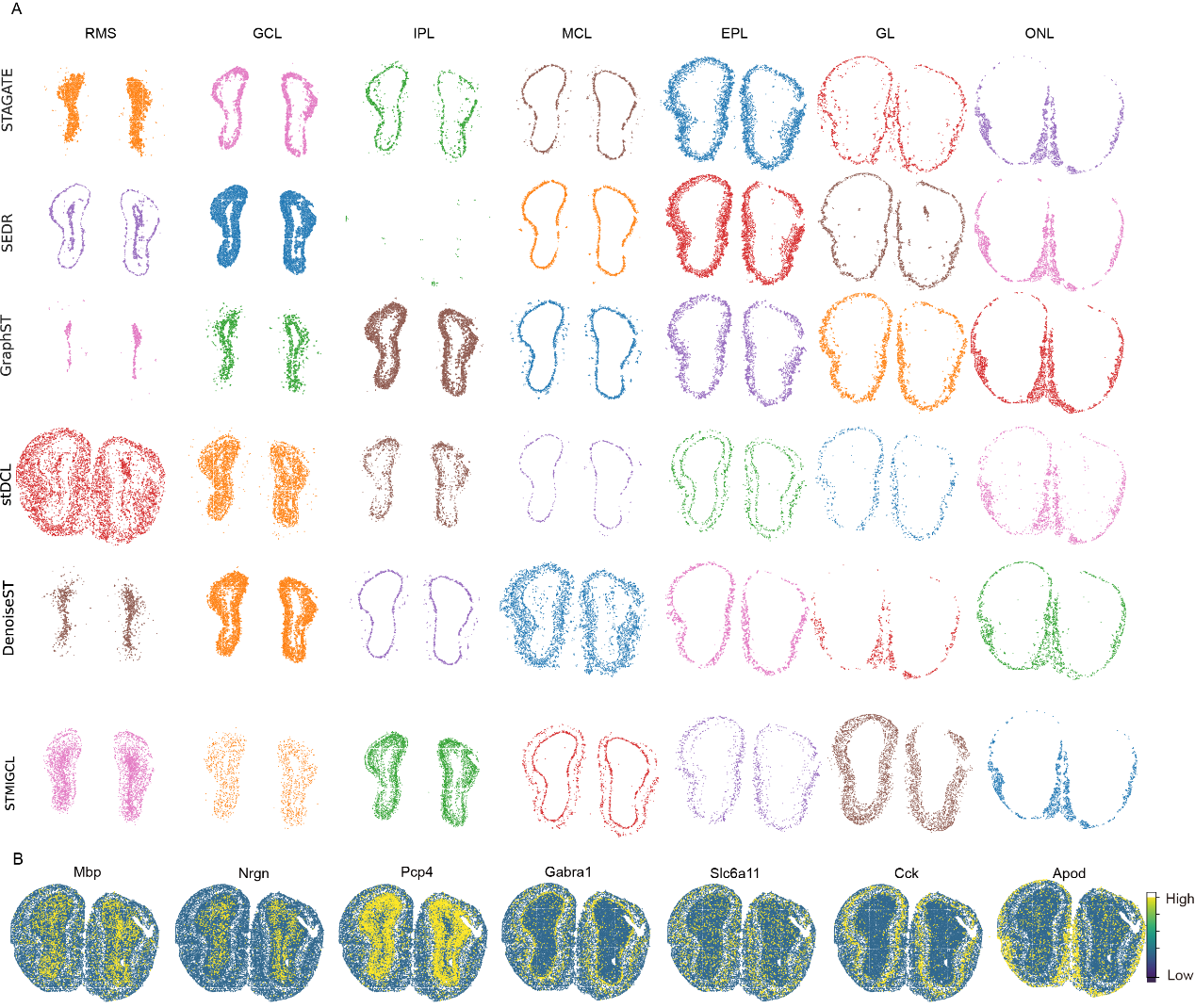
**

Figure S4. Clustering performance of benchmark experiments on the mouse olfactory bulb dataset. (A) Clustering results of the baseline method are displayed by layer. (B) Marker genes for each layer of the mouse olfactory bulb dataset

**Supplementary File 6：Clustering performance of benchmark experiments on multi-slice datasets**

**
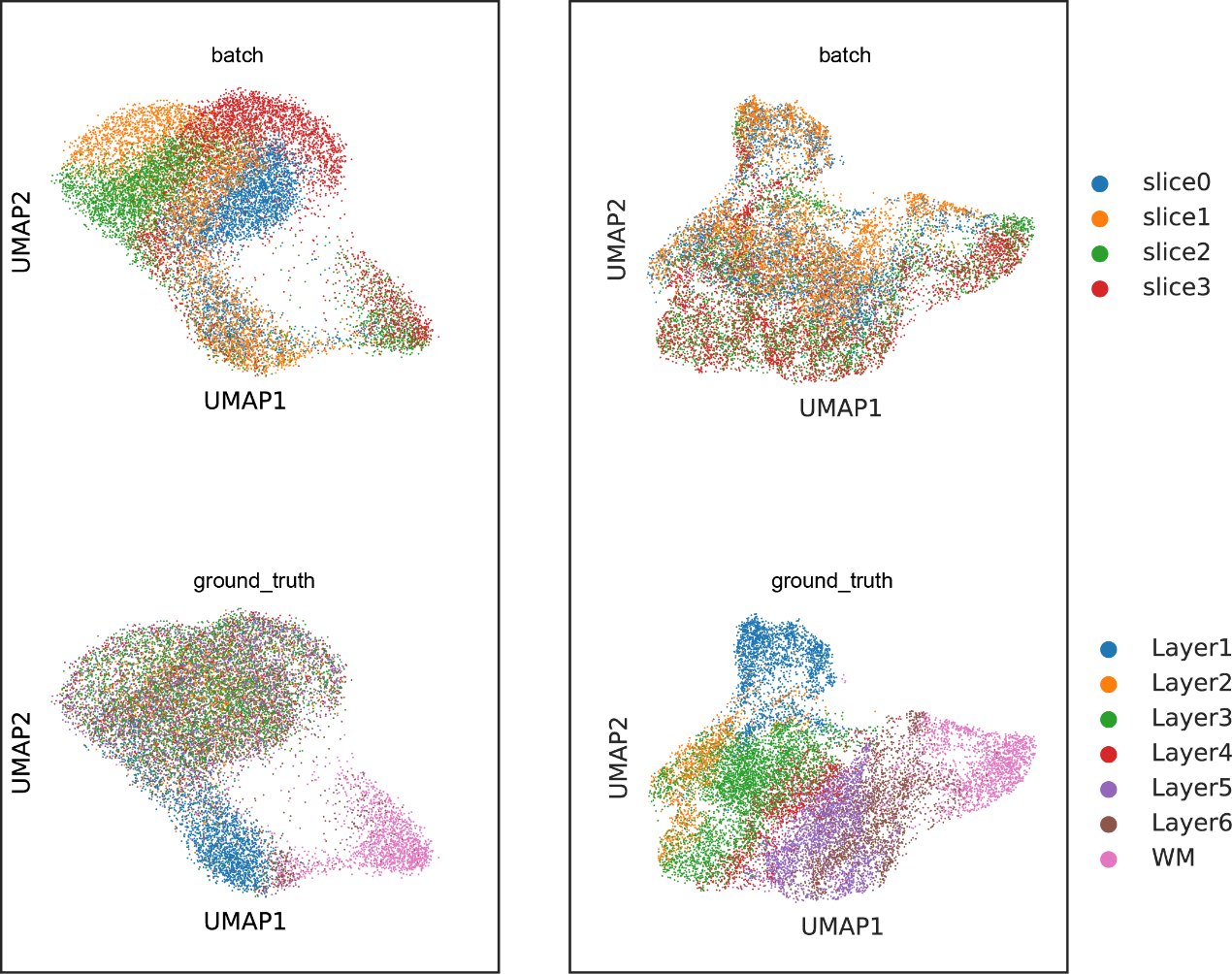
**

Figure S5. UMAP visualizations of cross-donor integration before and after SLGCA processing. The left panel shows the raw data, with batch-colored UMAP (top) and ground-truth labels (bottom). Strong batch effects are evident, and the spatial domains cannot be clearly distinguished. The right panel shows the data after SLGCA integration. Batch effects are largely mitigated, and the ground-truth spatial domains are more clearly separated, demonstrating that SLGCA effectively aligns slices across donors while preserving biologically meaningful structures.

**
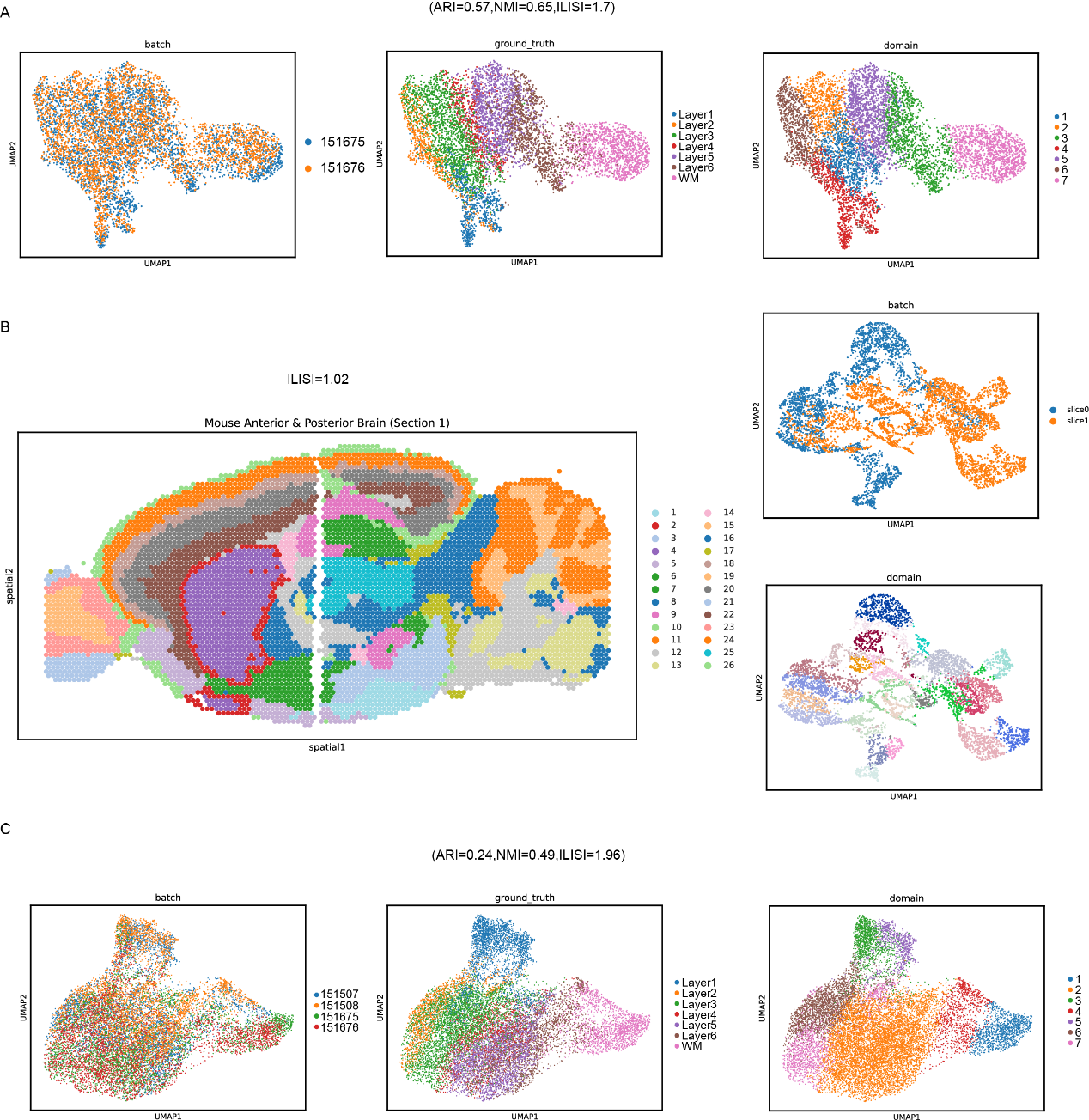
**

Figure S6. Clustering performance of GraphST on the multi-slice dataset. (A) Clustering performance on vertical slices. (B) Clustering performance on horizontal slices. (C) Clustering performance on slices from different sources.

**
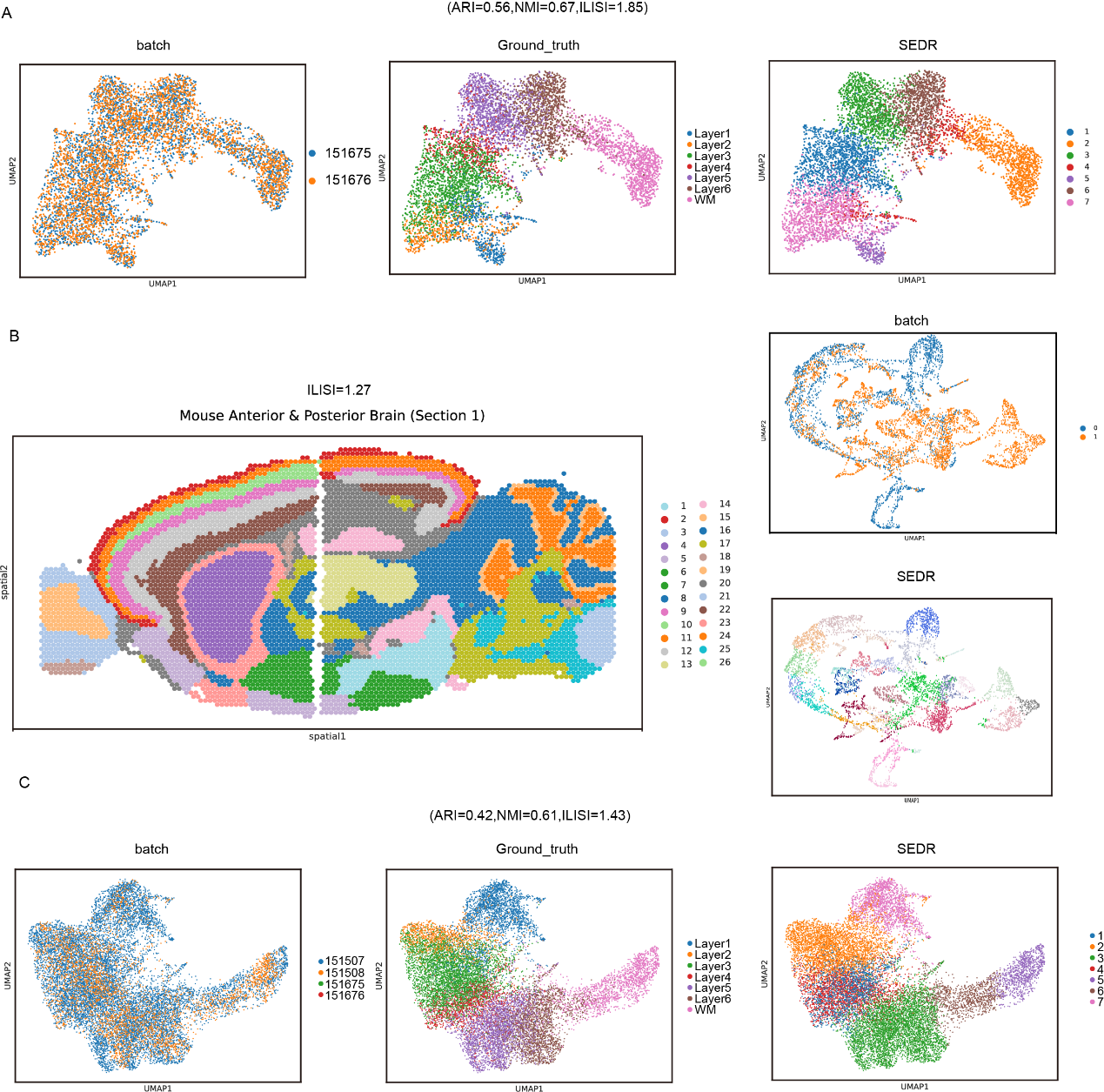
**

Figure S7. Clustering performance of SEDR on the multi-slice dataset. (A) Clustering performance on vertical slices. (B) Clustering performance on horizontal slices. (C) Clustering performance on slices from different sources.

**
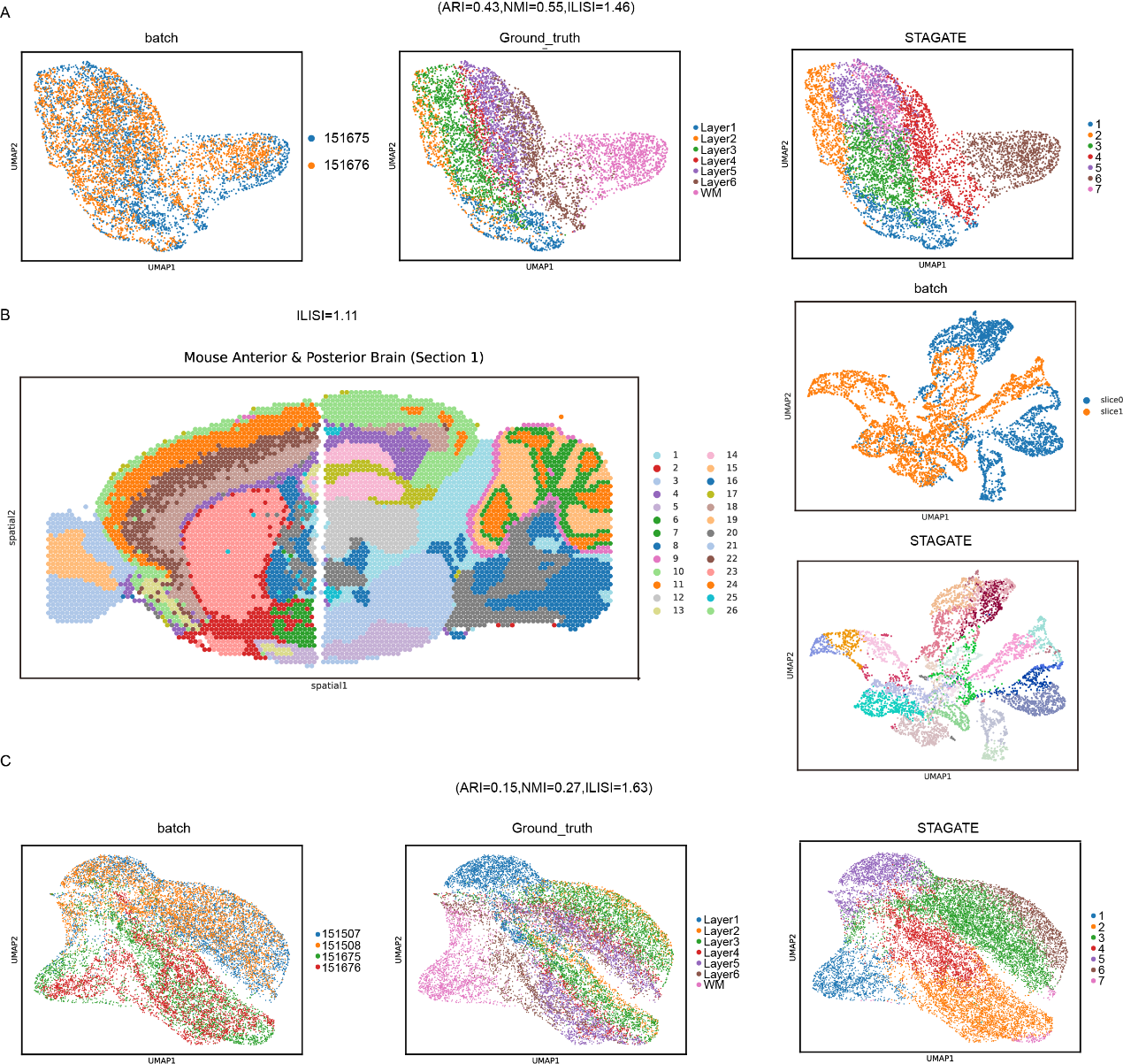
**

Figure S8. Clustering performance of STAGATE on the multi-slice dataset. (A) Clustering performance on vertical slices. (B) Clustering performance on horizontal slices. (C) Clustering performance on slices from different sources.

**Supplementary File 7：Enrichment analysis results of the human breast cancer dataset**

In this part of the study, we analyzed the differentially expressed genes using the online tool Metascape. Metascape provides GO enrichment, KEGG pathway analyses, and various additional pathways for user selection (for details, see https://metascape.org/). We also provide the lists of differentially expressed genes identified by SLGCA in this study; details can be found at https://github.com/luxin-heart/SLGCA.**
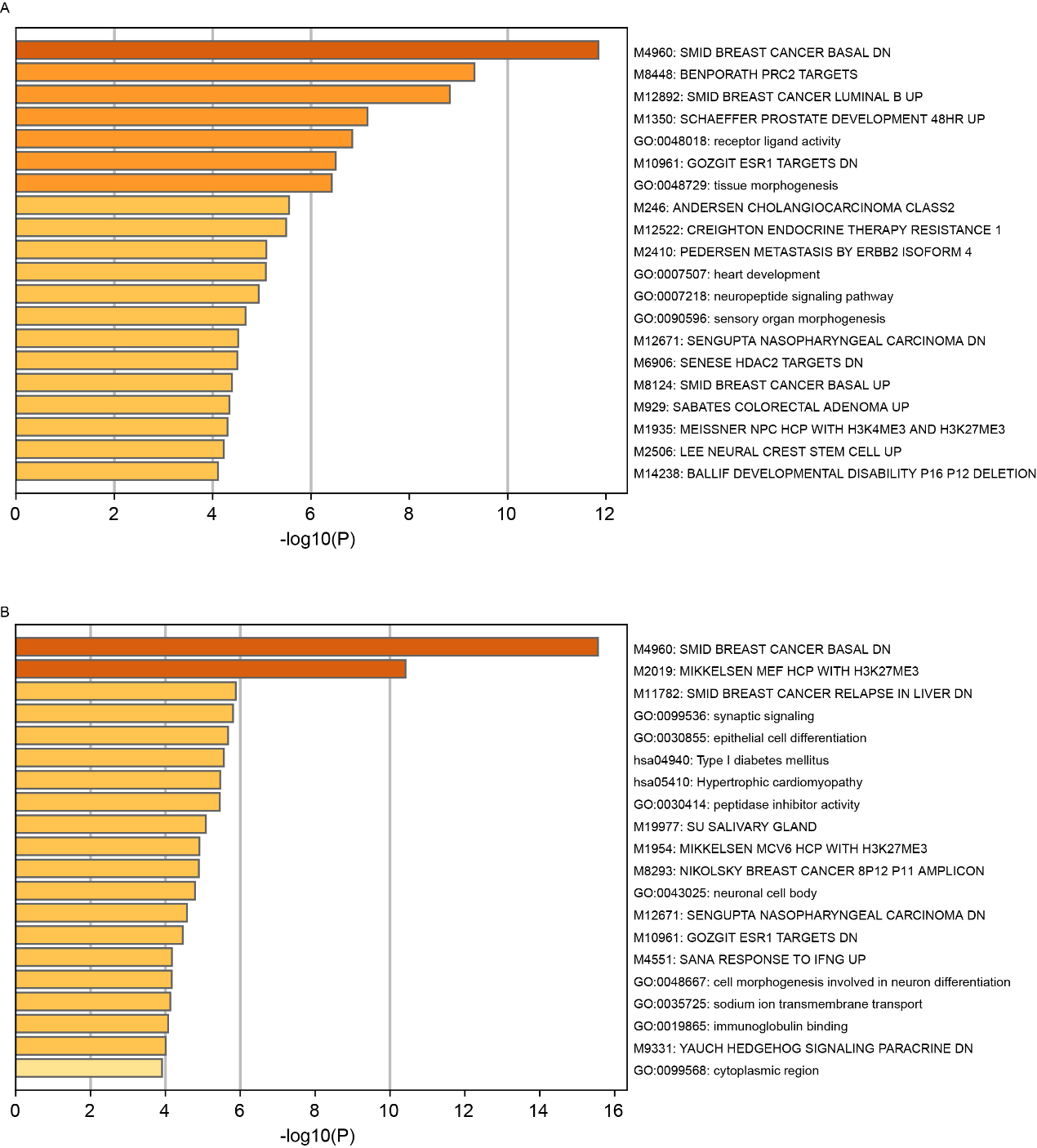
**

Figure S9. Enrichment analysis results of the human breast cancer dataset. (A) Enrichment analysis results of up-regulated genes in cluster 5. (C) Enrichment analysis results of up-regulated genes in cluster 10.


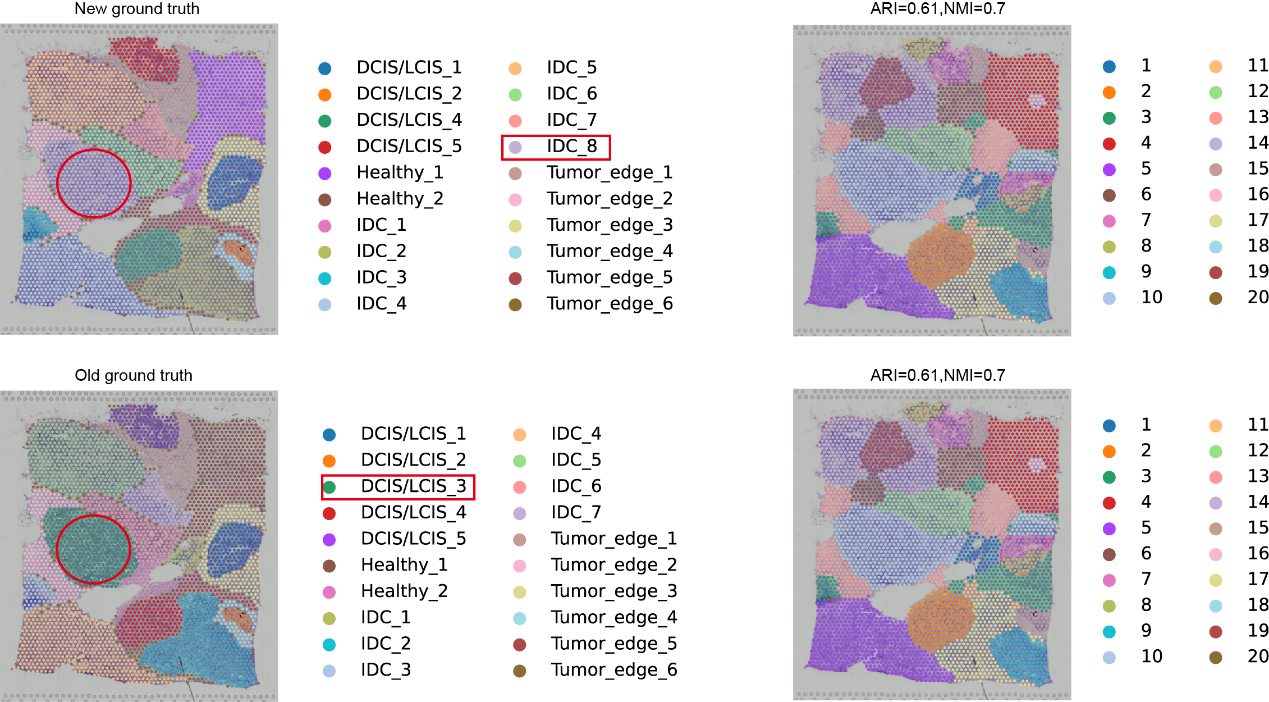


Figure S10. Clustering results of SLGCA under different labels in the BRCA dataset

**Supplementary File 8：Evaluation metrics for SLGCA**

In our study, we used two evaluation metrics to measure the clustering performance of the model: Adjusted Random Index (ARI) and Normalized Mutual Information (NMI).

ARI is a corrected-for-chance version of the Rand Index, commonly used to evaluate the similarity between two clustering results. Its value ranges from −1 to 1, where a score close to 1 indicates a high degree of agreement between the clustering and the reference labels, suggesting good clustering performance. A value of 0 implies a result equivalent to random assignment, while negative values indicate systematic disagreement. The ARI is calculated as follows:

$ARI\left( U,V \right)=\frac{RI\left( U,V \right)\mathbb{-E}\left[ RI\left( U,V \right) \right]}{max\left( RI\left( U,V \right)\mathbb{-E}\left[ RI\left( U,V \right) \right] \right)}$ (1)

where $\mathbb{E}\left[ \cdot\right]$ denotes expectation, and $RI\left( \cdot\right)$ represents the similarity between two clustering results. The calculation formula is shown below:

$RI\left( U,V \right)=\frac{TP+TN}{TP+TN+FP+FN}$ (2)

NMI measures the clustering quality by computing the normalized mutual information between the predicted and ground truth labels. Its value ranges from 0 to 1, where a higher NMI indicates greater similarity between the predicted clustering and the true class labels. The definition of NMI is as follows:

$NMI\left( U,V \right)=\frac{MI\left( U,V \right)}{\sqrt{H\left( U \right)H\left( V \right)}}$ (3)

here, *U* and *V* correspond to the true labels and clustering results. $MI\left( \cdot\right)$ denotes mutual information, and $H\left( \cdot\right)$ denotes entropy.

In the slice alignment experiments, we introduced the ILISI (Inverse Local Inverse Simpson's Index) metric to evaluate the quality of spatial transcriptomics (ST) data integration. The ILISI value typically ranges from 1 to $B$, where $B$ denotes the total number of slices to be aligned. A higher ILISI score indicates better mixing of cells from different slices within the embedded space, reflecting a more effective integration. The ILISI for the $i$-th cell is defined as:

${ILISI}_{i}=\left( \sum_{b=1}^{B} \left( {p_{i}}^{(b)} \right)^{2} \right)^{-1}$ (4)

where $i$ denotes the index of the cell; ${p_{i}}^{(b)}$ represents the proportion of cells from slice $b$ in the neighborhood of cell $i$; and $B$ is the total number of slices.

The overall ILISI score is then computed by averaging across all $N$ cells:

$ILISI = \frac{1}{N}\sum_{i=1}^{N} {ILISI}_{i}$ (5)

In addition to the above metrics, we further evaluate the clustering performance without labels using three unsupervised spatial metrics: the silhouette coefficient (SC), PAS, and CHAOS. SC measures cluster compactness and separation, with higher values indicating better clustering quality. PAS (Percentage of Abnormal Spots) quantifies the proportion of spatially adjacent spots assigned to different clusters, where lower values reflect higher spatial consistency. CHAOS (Clustering Heterogeneity Across Original Spatial-neighborhoods) assesses the degree of clustering heterogeneity within spatial neighborhoods, with lower values indicating stronger spatial continuity.

**Supplementary File 9：Robustness and efficiency analysis of SLGCA**

To assess the contribution of each component within the SLGCA framework, we conducted a series of ablation experiments. Specifically, we removed the local-level contrastive learning module(Without_Loc) in the first set of experiments. In the second set, we excluded the global-level contrastive learning module(Without_Glo). Both contrastive learning modules were removed in the third set, leaving only the core GCAE backbone and the inner product decoder (Without_Clu). In the first case (Without_Loc), the SLGCA model achieved an average ARI of 0.52 and a median of 0.53, while the average NMI was 0.65 with a median of 0.66. In the second case (Without_Glo), the average ARI was 0.53 with a median of 0.51, and the average and median NMI values were 0.65. In the third case (Without_Clu), the average ARI decreased to 0.49 with a median of 0.48, and the average and median NMI values dropped to 0.60 and 0.58, respectively. These results demonstrate that both the local and global contrastive learning modules play a significant role in enhancing the performance of the SLGCA model. When only one of the modules is removed (Without_Loc or Without_Glo), the ARI and NMI scores show a slight decline but remain relatively high overall. However, model performance degrades noticeably when both modules are removed simultaneously (Without_Clu), with substantial reductions in ARI and NMI. This highlights the critical importance of the contrastive learning components in improving clustering accuracy and representation quality in SLGCA. See Tables S5-S7 for details.

Table S5. Without_Loc

| **Slice** | **ARI** | **NMI** |
| --- | --- | --- |
| 151507 | 0.47 | 0.66 |
| 151508 | 0.51 | 0.67 |
| 151509 | 0.36 | 0.57 |
| 151510 | 0.33 | 0.53 |
| 151669 | 0.47 | 0.6 |
| 151670 | 0.46 | 0.67 |
| 151671 | 0.64 | 0.74 |
| 151672 | 0.65 | 0.72 |
| 151673 | 0.67 | 0.74 |
| 151674 | 0.62 | 0.72 |
| 151675 | 0.57 | 0.68 |
| 151676 | 0.55 | 0.65 |
| Mean | 0.52 | 0.65 |
| Median | 0.53 | 0.66 |

Table S6. Without_Glo

| **Slice** | **ARI** | **NMI** |
| --- | --- | --- |
| 151507 | 0.44 | 0.65 |
| 151508 | 0.48 | 0.65 |
| 151509 | 0.37 | 0.57 |
| 151510 | 0.45 | 0.61 |
| 151669 | 0.47 | 0.59 |
| 151670 | 0.44 | 0.55 |
| 151671 | 0.64 | 0.74 |
| 151672 | 0.65 | 0.72 |
| 151673 | 0.66 | 0.74 |
| 151674 | 0.62 | 0.71 |
| 151675 | 0.57 | 0.68 |
| 151676 | 0.54 | 0.64 |
| Mean | 0.53 | 0.65 |
| Median | 0.51 | 0.65 |

Table S7. Without_Clu

| **Slice** | **ARI** | **NMI** |
| --- | --- | --- |
| 151507 | 0.44 | 0.59 |
| 151508 | 0.44 | 0.57 |
| 151509 | 0.33 | 0.54 |
| 151510 | 0.43 | 0.57 |
| 151669 | 0.44 | 0.53 |
| 151670 | 0.39 | 0.5 |
| 151671 | 0.6 | 0.68 |
| 151672 | 0.6 | 0.67 |
| 151673 | 0.61 | 0.69 |
| 151674 | 0.58 | 0.68 |
| 151675 | 0.54 | 0.65 |
| 151676 | 0.51 | 0.58 |
| Mean | 0.49 | 0.6 |
| Median | 0.48 | 0.58 |

In addition, we also conducted an ablation experiment on the topological decoder. The experimental results show that the topological decoder also plays an essential role in the entire model architecture. The following table shows the specific clustering results.

Table S8. Without_Topological Decoder

| **Slice** | **ARI** | **NMI** |
| --- | --- | --- |
| 151507 | 0.44 | 0.65 |
| 151508 | 0.5 | 0.67 |
| 151509 | 0.37 | 0.57 |
| 151510 | 0.47 | 0.61 |
| 151669 | 0.47 | 0.59 |
| 151670 | 0.46 | 0.56 |
| 151671 | 0.64 | 0.74 |
| 151672 | 0.65 | 0.72 |
| 151673 | 0.63 | 0.73 |
| 151674 | 0.6 | 0.71 |
| 151675 | 0.57 | 0.68 |
| 151676 | 0.6 | 0.7 |
| Mean | 0.53 | 0.66 |
| Median | 0.53 | 0.68 |

Meanwhile, to verify the robustness of SLGCA and the impact of hyperparameters on model performance, we conducted a hyperparameter analysis of SLGCA. Based on previous studies, we selected two key parameters: the number of highly variable genes and the number of neighbors. Specifically, the range of the number of highly variable genes is (1000, 2000, 3000, 4000, 5000), and the range of neighbors is (3, 4, 5, 6, 7). The experimental results show that the model performs best when the number of highly variable genes is 4000 and the number of neighbors is 4. Under other parameter conditions, the model’s performance will decline to varying degrees. See Tables S7 and S8 for details.

Table S9. The number of highly variable genes

| **The number of highly variable genes** | **ARI** | **NMI** |
| --- | --- | --- |
| 1000 | 0.46 | 0.61 |
| 2000 | 0.58 | 0.67 |
| 3000 | 0.6 | 0.69 |
| 4000 | 0.63 | 0.71 |
| 5000 | 0.57 | 0.65 |

Table S10. The number of neighbors

| **The number of neighbors** | **ARI** | **NMI** |
| --- | --- | --- |
| 3 | 0.55 | 0.65 |
| 4 | 0.63 | 0.71 |
| 5 | 0.58 | 0.69 |
| 6 | 0.58 | 0.67 |
| 7 | 0.57 | 0.67 |

In addition, we investigated the impact of loss function weights on model performance using the DLPFC dataset. Specifically, we fixed α at 10 and varied the values of β and γ within the range of (0.3, 0.5, 0.7, 0.9). The experimental results showed that the model achieved the best performance when both β and γ were set to 0.5. Detailed results for all settings are provided in *Loss parameter analysis.csv* at https://github.com/luxin-heart/SLGCA. Based on this setting, we further conducted a hyperparameter search for α, testing values in (0.5, 1, 3, 5, 7, 10, 15, 20). The results, summarized in the table below, indicate that the model achieved optimal performance when α was set to 10.

Table S11. Clustering indicators under different α parameters

| **Parameter α** | **Mean ARI** | **Median ARI** | **Mean NMI** | **Median NMI** |
| --- | --- | --- | --- | --- |
| 0.5 | 0.53 | 0.52 | 0.66 | 0.66 |
| 1 | 0.51 | 0.48 | 0.65 | 0.64 |
| 3 | 0.53 | 0.52 | 0.66 | 0.64 |
| 5 | 0.53 | 0.5 | 0.66 | 0.64 |
| 7 | 0.55 | 0.54 | 0.67 | 0.66 |
| **10** | **0.63** | **0.65** | **0.71** | **0.72** |
| 15 | 0.55 | 0.54 | 0.67 | 0.68 |
| 20 | 0.54 | 0.54 | 0.66 | 0.66 |

To further understand the running efficiency of SLGCA, we evaluated the runtime performance and memory usage of SLGCA on two representative datasets: the DLPFC dataset generated using the 10× Visium platform, and the mouse olfactory bulb dataset generated using the Stereo-seq platform, which contains the largest number of cells among all datasets in our study. It is worth noting that STMIGCL was excluded from this evaluation due to its excessively long runtime requirements. The results are shown in Tables S1 and S2. The results show that SLGCA achieved the shortest runtime and the lowest memory consumption on the DLPFC dataset. However, when applied to the mouse olfactory bulb dataset generated using the Stereo-seq platform, SLGCA exhibited longer runtimes and higher memory usage, indicating that its performance requires further improvement when handling large-scale datasets such as those generated by Stereo-seq.

Table S12. The running efficiency of SLGCA and baseline methods on the DLPFC dataset

| **Method** | **Running Time(second)** | **CPU memory(GB)** |
| --- | --- | --- |
| SLGCA | 285 | 3.83 |
| GraphST | 323 | 9.65 |
| SEDR | 432 | 5.22 |
| STAGATE | 583 | 4.28 |
| stDCL | 593 | 8.53 |
| DenoiseST | 335 | 4.32 |

Table S13. The running efficiency of SLGCA and baseline methods on the mouse olfactory bulb dataset

| **Method** | **Running Time(second)** | **CPU memory(GB)** |
| --- | --- | --- |
| SLGCA | 156 | 6.44 |
| GraphST | 33 | 2.7 |
| SEDR | 110 | 1.6 |
| STAGATE | 103 | 2.1 |
| stDCL | 277 | 6.34 |
| DenoiseST | 220 | 12.4 |

We compared several alternative methods to examine further the effect of graph construction strategies on model performance. In addition to the commonly adopted approach based on spatial coordinates combined with K-nearest neighbors (KNN), we evaluated three other strategies: constructing graphs using cosine similarity of gene expression profiles, Pearson correlation coefficients, and the Shared Nearest Neighbors (SNN) method. Benchmark experiments conducted on the DLPFC dataset demonstrated that the spatial-based KNN strategy achieved the best overall performance, as it effectively captures both the spatial organization and the underlying gene expression patterns. Specific indicators can be found in the table below.

Table S14. Performance of SLGCA on the DLPFC dataset under different composition methods

| **Graph construction** | **Mean ARI** | **Median ARI** | **Mean NMI** | **Median NMI** |
| --- | --- | --- | --- | --- |
| KNN | 0.63 | 0.65 | 0.71 | 0.72 |
| Cosine similarity | 0.46 | 0.44 | 0.59 | 0.57 |
| Pearson correlation | 0.5 | 0.47 | 0.62 | 0.61 |
| SNN | 0.53 | 0.54 | 0.64 | 0.64 |

We also evaluated the downstream clustering performance of SLGCA using four commonly applied algorithms: Mclust, Louvain, Leiden, and K-means. The results show that Mclust achieved the best clustering performance, whereas K-means exhibited relatively inferior results.

Table S15. Performance of SLGCA on the DLPFC dataset under different Clustering algorithms

| **Clustering algorithms** | **Mean ARI** | **Median ARI** | **Mean NMI** | **Median NMI** |
| --- | --- | --- | --- | --- |
| Mclust | 0.63 | 0.65 | 0.71 | 0.72 |
| K-means | 0.4 | 0.4 | 0.56 | 0.56 |
| Leiden | 0.48 | 0.45 | 0.61 | 0.61 |
| Louvain | 0.46 | 0.46 | 0.6 | 0.59 |

Finally, we conducted experiments using the learned intermediate embeddings $Z$ as input to evaluate model performance. The results show that, although using $Z$ yields slightly lower performance compared to using the reconstructed expression profiles $H$, it still achieves competitive results, further demonstrating the robustness of our framework. Specifically, when using $Z$, both the mean and median ARI reached 0.55, while the mean and median NMI were 0.66. The overall scores are summarized in the table below:

Table S16. Clustering performance of SLGCA using embeddings as input vs. using reconstruction matrices as input on the DLPFC dataset.

| **Slice** | **ARI(Z)** | **ARI(H)** | **NMI(Z)** | **NMI(H)** |
| --- | --- | --- | --- | --- |
| 151507 | 0.51 | 0.57 | 0.67 | 0.71 |
| 151508 | 0.55 | 0.55 | 0.64 | 0.69 |
| 151509 | 0.41 | 0.53 | 0.6 | 0.69 |
| 151510 | 0.52 | 0.55 | 0.61 | 0.66 |
| 151669 | 0.56 | 0.66 | 0.66 | 0.68 |
| 151670 | 0.47 | 0.66 | 0.55 | 0.65 |
| 151671 | 0.67 | 0.65 | 0.75 | 0.74 |
| 151672 | 0.68 | 0.77 | 0.73 | 0.75 |
| 151673 | 0.58 | 0.66 | 0.69 | 0.75 |
| 151674 | 0.58 | 0.65 | 0.68 | 0.74 |
| 151675 | 0.53 | 0.63 | 0.64 | 0.72 |
| 151676 | 0.54 | 0.66 | 0.67 | 0.73 |
| Mean | 0.55 | 0.63 | 0.66 | 0.71 |
| Median | 0.55 | 0.65 | 0.66 | 0.72 |

**Supplementary File** **10：****Detailed description of the downstream application design process**

**Spatial domain identification**

Following model training, the reconstructed gene expression matrix was used for dimensionality reduction via principal component analysis (PCA). Subsequently, spatial domain identification was performed using the Mclust clustering algorithm implemented in **R** (v4.2.0). Spatial visualization of the clustering results was conducted using the sc.pl.spatial() function from the **Scanpy** package(v1.11.1). Specifically, spatial domains were visualized by setting the color parameter to either the ground truth (manually annotated tissue layers) or predicted cluster labels. Additionally, marker gene expression patterns were visualized by specifying the corresponding gene names in the same function.

**Differential gene expression analysis**

To identify key regulatory genes distinguishing different spatial domains, we performed differential expression analysis using the sc.tl.rank_genes_groups() function in **Scanpy** (v1.11.1), employing t-test as the statistical method. Genes were considered significantly differentially expressed if they met the following criteria: p-value < 0.05 (statistical significance), |log₂ fold change|> 2 (biological significance). The resulting differentially expressed genes (DEGs) were retained for subsequent functional enrichment analysis.

**Functional enrichment analysis**

Functional enrichment analysis of the DEGs was performed using **Metascape** (https://metascape.org/). Enrichment was conducted across multiple annotation categories, including Gene Ontology (GO): biological processes (GO:_BP), molecular functions (GO:_MF), and cellular components (GO:_CC); Kyoto Encyclopedia of Genes and Genomes (KEGG) pathways and Chemical and Genetic Perturbations (CGP) pathways. These analyses facilitated the identification of functional themes and biological pathways associated with spatially regulated genes.

In addition, the **ClusterProfiler** R package was also used to perform Gene Ontology (GO) and KEGG enrichment analyses via the functions “enrichGO” and “enrichKEGG”. Enrichment analysis was conducted using this method for the liver cancer dataset analyzed in this study.

**Cell type annotation**

After clustering the dataset, cell types were annotated using the **SingleR** method from the R package. This approach assigns cell types based on marker genes associated with the dataset.

**Trajectory analysis**

We applied **Monocle2** for trajectory inference. Highly variable genes were selected for ordering, followed by dimensionality reduction with DDRTree to reconstruct a branched trajectory. Cells were ordered in pseudotime with a defined root state, and branch-specific genes were identified using BEAM.

**Supplementary File 11：Comparison with other contrastive learning methods**

To further highlight the innovations of SLGCA and distinguish it from other contrastive learning-based approaches, we compared two representative methods, GraphST and stGCL. We conducted a detailed analysis across three key aspects: model architecture, contrastive learning mechanism, and loss function design. This comparative study underscores the unique advantages and innovations of SLGCA.

1. **Model Architecture**:

GraphST employs an autoencoder architecture constructed using Graph Convolutional Networks (GCNs).

stGCL uses an autoencoder architecture based on Graph Attention Networks (GATs).

SLGCA, while using a GCN-based autoencoder architecture, incorporates an additional topology decoder based on an inner product mechanism to reconstruct the spatial adjacency matrix. This topology decoder makes SLGCA more robust in capturing spatial relationships.

1. **Contrastive Learning Mechanism**:

GraphST adopts a contrastive learning approach inspired by the Deep Graph Infomax (DGI) framework, focusing on a local-to-global contrastive learning mechanism. It extracts intermediate embeddings and uses a readout function to enhance the quality of these embeddings through contrastive learning.

Similarly, stGCL is influenced by the DGI framework. It also employs a readout function to generate graph-level summaries from intermediate embeddings, which are optimized using a binary cross-entropy-based contrastive learning loss. Both methods primarily rely on a local-to-global contrastive learning paradigm.

SLGCA, on the other hand, employs a dual-channel contrastive learning mechanism that focuses on both local-to-local and global-to-global levels of information:

Local-to-local contrastive learning: SLGCA considers spatial neighbor information when constructing positive and negative sample pairs. Specifically, when creating positive sample pairs, it not only matches the anchor point with its corresponding point in the contrastive view but also incorporates the neighbors of the anchor point in the original view and the neighbors of the corresponding point in the contrastive view.

Global-to-global contrastive learning: SLGCA uses a readout function to generate global-level summaries. However, unlike GraphST and stGCL, SLGCA does not directly perform contrastive learning on these summaries. Instead, it constructs a cosine similarity matrix between the two global summaries, capturing the relationships among all samples at a global scale. An ideal cosine similarity matrix should resemble a diagonal matrix, as this reflects higher similarity between positive samples. SLGCA optimizes the cosine similarity matrix via contrastive learning to make it more diagonal, thereby enhancing global information learning.

1. **Loss Function**:

GraphST and stGCL use a combination of reconstruction loss and contrastive learning loss in their objectives. Their contrastive learning loss is based on a binary cross-entropy formulation.

However, SLGCA incorporates three components into its loss function: Reconstruction loss, which includes gene expression matrix reconstruction and adjacency matrix reconstruction losses, Local contrastive learning loss, and Global contrastive learning loss. Specifically, the local contrastive learning loss is formulated based on mutual information maximization, whereas the global contrastive learning loss is constructed using the mean squared error (MSE) loss.

**Supplementary File 12：Global representation using Pearson correlation**

For the global-level contrastive learning mechanism, we further conducted an additional experiment. Specifically, instead of applying a readout function, we computed the Pearson correlation matrix between two intermediate embeddings and then constructed the loss function using this Pearson correlation matrix together with the diagonal matrix, following the formulation in Equation (15) of the main text. We performed a benchmark experiment on the DLPFC dataset, and the detailed results are summarized in the table below:

Table S17. Clustering performance of SLGCA using Pearson correlation matrix on the DLPFC dataset

| **Slice** | **ARI** | **NMI** |
| --- | --- | --- |
| 151507 | 0.44 | 0.65 |
| 151508 | 0.46 | 0.63 |
| 151509 | 0.37 | 0.57 |
| 151510 | 0.46 | 0.64 |
| 151669 | 0.47 | 0.6 |
| 151670 | 0.45 | 0.56 |
| 151671 | 0.64 | 0.74 |
| 151672 | 0.65 | 0.72 |
| 151673 | 0.66 | 0.74 |
| 151674 | 0.62 | 0.72 |
| 151675 | 0.56 | 0.69 |
| 151676 | 0.62 | 0.71 |
| Mean | 0.53 | 0.66 |
| Median | 0.52 | 0.67 |
